# In silico screening for safe and potent essential oils against mosquito neural targets

**DOI:** 10.1186/s12983-026-00620-7

**Published:** 2026-06-13

**Authors:** Mohamed Nagy, Esraa Mansour, Hanaa Elbrense

**Affiliations:** Department of Zoology, Faculty of Science, Tanta University, Tanta, 31527 Egypt

**Keywords:** Essential oils, Mosquito control, In silico screening, Molecular docking, *Culex pipiens*, *Matricaria chamomilla*

## Abstract

**Background:**

Mosquito-borne diseases remain a major global health challenge, highlighting the need for eco-friendly alternatives to conventional chemical insecticides. This study aimed to evaluate a tiered in silico screening framework to prioritize plant-derived essential oils as potential sustainable mosquito control agents.

**Results:**

A large-scale virtual screening of 5,183 essential oils was conducted, followed by toxicity-based filtering, yielding 1,345 low-toxicity candidates. Subsequent molecular docking against neural targets of *Culex pipiens* identified 51 top-priority oils. Among these, German chamomile (*Matricaria chamomilla*) consistently appeared across multiple chemotypes. The Egyptian German chamomile essential oil was selected for experimental validation. Chemical analysis revealed sesquiterpenes, particularly bisabolol oxide derivatives and β-farnesene, as major constituents. Docking results demonstrated strong binding affinities toward multiple neural targets. Larvicidal bioassays showed 100% mortality at 400 ppm after 24 h, accompanied by significant behavioral, morphological,and histopathological abnormalities. Neurochemical assays indicated increased acetylcholinesterase activity, alongside decreased γ-aminobutyric acid levels, suggesting disruption of neural signaling.

**Conclusions:**

This study demonstrates the effectiveness of in silico screening in identifying safe and potent plant-based mosquito control agents. German chamomile essential oil emerges as a promising eco-friendly candidate for sustainable vector management, warranting further field-based validation.

**Supplementary Information:**

The online version contains supplementary material available at 10.1186/s12983-026-00620-7.

## Background

Mosquito-borne diseases remain a major public health challenge worldwide, with several pathogens transmitted by different mosquito species posing significant threats to healthcare [1]. Among the medically important species is *Culex pipiens* (Diptera: Culicidae), commonly known as the common house mosquito. It transmits diseases such as Lymphatic filariasis, West Nile fever, and St. Louis encephalitis [2]. Conventional chemical insecticides have been the primary means of mosquito control for decades; however, their effectiveness has declined due to the rapid development of resistance in mosquito populations [3]. Moreover, their extensive use raises environmental and human health concerns [4], highlighting the need for alternative strategies that are both effective and environmentally sustainable. Plant-derived natural products have attracted considerable interest as potential mosquito control agents, with essential oils (EOs) emerging as particularly promising due to their insecticidal and repellent properties [5–7]. The bioactivity of EOs is largely attributed to their chemical constituents, which act via multiple mechanisms, including disruption of the insect cuticle, interference with the respiratory system, and modulation of the nervous system [8]. Neuroactive compounds, in particular, can modulate neurotransmission by interacting with critical neural targets, including acetylcholinesterase (AChE), γ-aminobutyric acid receptors (GABA_R_), voltage-gated sodium channels (VGSC), and octopamine receptor (OAR) [9]. Despite the promising mosquitocidal potential of EOs, conventional screening approaches remain time-consuming, costly, and labor-intensive, limiting the rapid identification of effective candidates. In silico methods offer a scalable alternative by enabling high-throughput screening, prediction of target interactions, assessment of physicochemical properties, and early toxicity evaluation, thereby facilitating more focused experimental validation. In this context, the present study introduces a large-scale in silico pipeline for identifying promising EO candidates with larvicidal activity against *Cx. pipiens*. The workflow integrates virtual screening of 5,183 EOs, vertebrate toxicity filtering, and molecular docking against key mosquito neural targets, enabling the prioritization of candidates with high predicted mosquitocidal activity and low vertebrate toxicity. Furthermore, selected candidate EOs identified were subjected to subsequent experimental validation to support the biological relevance of the predictions. This study offers an efficient framework for accelerating the discovery of plant-derived mosquito control agents.

## Materials and methods

### Data collection and compound identification

A large dataset of EOs and their chemical constituents was collected from three open-access databases: sCentIndb (Essential Oil Chemical Profiles of Medicinal Plants of India) [10], EOUdb (Essential Oil Database) (https://essentialoils.org/about), and AromaDb (Plant Aroma Molecules Database) [11]. All databases were accessed in July 2025. For each oil, the complete chemical profile, including both major and minor components, was retrieved along with their reported relative abundances (%). Compound names were standardized, cross-validated, and mapped to their corresponding PubChem Compound Identifiers (CIDs) to ensure structural consistency across sources. The dataset was further curated by removing duplicate entries, correcting nomenclature inconsistencies, and excluding compounds lacking valid PubChem annotations.

### Toxicity prediction

Toxicity profiles for all curated phytochemical compounds were predicted using the ProTox-3.0 [12] web server, an advanced in silico platform for small-molecule toxicity assessment based on machine learning and chemical similarity modeling. Canonical SMILES structures for all compounds were retrieved from PubChem [13] and submitted to ProTox-3.0 for toxicity prediction. The platform generated multiple parameters for each compound, including acute oral toxicity values (LD₅₀, expressed mg·kg⁻¹), toxicity class, average structural similarity, and prediction accuracy, along with additional data for diverse toxicological endpoints such as organ toxicity, general toxicity effects, nuclear receptor and stress response pathways, molecular initiating events, metabolism features, and predicted toxicity targets. All predicted values were accompanied by corresponding confidence scores, providing quantitative confidence of model predictions. The resulting dataset served as the foundation for calculating compound- and oil-level toxicity indices described in Sect. "Toxicity scoring and oil-level aggregation".

### Toxicity scoring and oil-level aggregation

To quantify the toxicological potential of individual compounds and their corresponding EOs, a structured scoring framework was developed. This framework integrates acute toxicity, regulatory classification, and mechanistic endpoint information in a transparent and interpretable manner, suitable for high-throughput in silico screening.

Predicted LD₅₀ were transformed into a normalized acute toxicity score using a logistic function:

$$\eqalign{ & LD50\_score = 1/exp \cr & \left( {(lo{g_{10}}\left( {LD50} \right) - 3.3)/1.1} \right) + 1 \cr}$$

This transformation maps LD₅₀ values to the interval [0, 1], where higher scores indicate greater acute toxicity. The logistic form provides a smooth transition across relevant LD₅₀ values and limits the influence of extreme values. The midpoint of the function (log₁₀(LD₅₀) ≈ 3.3, corresponding to ~ 2000 mg·kg⁻¹) aligns with widely used regulatory thresholds in the Globally Harmonized System [14], where compounds above this value are generally considered to pose low acute hazard.

Categorical toxicity class information was incorporated via a class-based score:

$$\:Clas{s}_{score}={\left((6-TC)/5\right)}^{0.75}$$

where $\:TC$ is the ProTox-3.0 toxicity class (1 = most toxic; 6 = least toxic). This rescales the ordinal classes to a continuous range between 0 and 1, with higher values representing more hazardous classes. The exponent (0.75) was selected to slightly compress the differences among the most toxic classes (1–3) [15], as they represent similarly high hazard levels.

Mechanistic and organ-specific toxicity was quantified using a target-based score derived from 45 toxicity-related endpoints. Only predictions classified as Toxic/Active were contributed, weighted by the model-reported confidence

$$\:{\:C}_{t}=\:reported\_percentage/100$$

while Non-toxic/Inactive predictions contributed zero. The overall target-based score was then calculated as a weighted average:

$$\:Targets\_score\:=\:{\sum\:}_{t}\left({\omega\:}_{t}\times\:{C}_{t}\right)/{\sum\:}_{t}{\omega\:}_{t}$$

where ωₜ represents the toxicological importance of endpoint t. Higher weights were assigned to endpoints with strong regulatory and human health relevance (e.g., carcinogenicity, mutagenicity, cytotoxicity, and major organ toxicities), while moderate weights were used for endocrine disruption, oxidative stress pathways, and mitochondrial dysfunction. Metabolic and auxiliary targets (such as CYP isoforms) were assigned lower weights. The final compound-level toxicity score integrates acute toxicity, toxicity class, and target-based information:

$$\eqalign{ & Final\_Compound \_Toxicity\_Score \cr & = round(\left( {0.35 \times LD50\_score \times 10} \right) \cr & + \left( {0.20 \times Class\_score \times 10} \right) \cr & + \left( {0.45 \times Targets\_score \times 10} \right)) \cr}$$

This weighting reflects contemporary toxicological priorities: acute toxicity provides regulatory anchoring, class-based scores add categorical context, and mechanistic endpoints capture organ-specific and potentially chronic toxic effects. The final score is reported on a standardized 0–10 scale, where higher values indicate greater predicted toxicological concern.

Essential oil (EO) toxicity was calculated as an abundance-weighted average of constituent compound scores:

$$\eqalign{ & Essential\_Oil \_Toxicity\_Index \cr & = \left( {{\matrix{ \mathop \sum \nolimits_{i = 1}^n (Percentag{e_i} \hfill \cr \times Final\_Compound\_Toxicity\_Scor{e_i} \hfill \cr} \over {\mathop \sum \nolimits_{i = 1}^n Percentag{e_i}}}} \right) \cr}$$

where Percentage_i_ represents the relative abundance of compound *i* within the oil. Normalization was performed using the sum of the identified compounds percentages rather than assuming a 100% composition, accounting for incomplete chemical characterization. This index provides an interpretable measure of overall toxicological concern, with higher values indicating EOs dominated by more hazardous constituents.

### Target and ligand preparation for docking analysis

Four mosquito neural protein targets were selected for molecular docking: (1) AChE (UniProt ID: Q86GC8; reference ligand: chlorpyrifos), (2) VGSC (A0A8D8AMN4; reference ligand: indoxacarb), (3) GABA_R_ (A0A8D8CG52; reference ligand: fipronil), and (4) OAR (A0A8D8H7Y3; reference ligand: amitraz). Three-dimensional structures were predicted using AlphaFold 3 [16], and potential ligand-binding sites were identified with P2Rank [17] employing the AlphaFold configuration model (-c alphafold), pocket probability scores were considered during binding-site selection to ensure confidence in the predicted cavities, and the predicted binding site residues were evaluated based on residue-level confidence scores (pLDDT) to ensure the binding site reliability used for docking analysis. Protein structures were prepared by removing water molecules, adding polar hydrogens, and assigning Gasteiger charges. Ligands were processed through a multi-step workflow: Canonical SMILES strings from the curated dataset were processed with Dimorphite-DL [18] to generate the most probable protonation states at a physiological pH range of 7.2–7.6. Each protomer was converted into an optimized three-dimensional structure using RDKit [19] toolkit with the ETKDG v3 [20] algorithm, followed by energy minimization with MMFF94 [21] or UFF [22] force fields, depending on atom-type compatibility. The lowest-energy conformers were converted to PDBQT format using Meeko [23] and organized for docking. Molecular docking simulations were performed using AutoDock Vina [24], with grids centered on predicted binding pockets. Binding free energy was calculated, and the results were analyzed to reveal affinity trends across the four mosquito neural targets. To validate the docking protocol, the reference ligand corresponding to each target protein was independently docked into its respective predicted binding site through 50 repeated docking runs. The resulting poses were compared, and the average root mean square deviation (RMSD) relative to the best-scoring pose was calculated to evaluate docking reproducibility and protocol stability. The docking poses and protein–ligand interactions were visualized using PyMOL [25].

### Computation of Essential Oil Potency Index

The relative insecticidal potency of each EO was quantified using an Integrated Mosquito Target Index (IMT), which integrates molecular binding strength, neural target coverage, and predicted safety into a composite score.

The relative abundance of each compound (*i*) within oil (*j*) was normalized as:

$$\eqalign{{f_{ij}} & = Precentag\,{e_{ij}}/\sum {i \in j} Precentag\,{e_{ij}}, \cr & ensuring\mathop \sum \limits_i {f_{ij}} = 1 \cr}$$

Docking-derived binding energies (*E*_*ik*_) between compound (*i*) and neural target (*k*) were converted into positive interaction strengths as: *A*_*ik*_ = $\:-$ *E*_*ik*_, Only interactions with *A*_*ik*_ ≥ 6 kcal·mol⁻¹ were retained; weaker interactions were set to zero. For each target (𝑘), interaction strengths were normalized using min–max scaling:

$$\eqalign{ {S_{ik}} & = ({A_{ik}} - {A_{min,k}})/ \cr & ({A_{max,k}} - {A_{min,k}}),{S_{ik}} \in \left[ {0,1} \right] \cr}$$

Where $\:{{A}_{min,k}\:\rm{and}\:A}_{max,k}$ represent the minimum and maximum binding strengths per target ($\:k)$, respectively.

The effective contribution of compound (*i*) to target (*k*) within oil (*j*) was defined as:

$$\:{e}_{ijk}={f}_{ij}\:\times\:{S}_{ik}$$

Target-specific coverage by oil (*j*) is calculated as:

$$\:Coverag\,{e}_{jk}=\:\sum\:_{i\in\:j}{e}_{ijk}$$

Target breadth was defined as the mean coverage across the four neural targets:

$$\:OilBreadt\,{h}_{j}=\:\frac{1}{4}\:\sum\:_{K=1}^{4}Coverag\,{e}_{jk}$$

To capture the intrinsic molecular binding strength of each oil, a raw binding score was computed as the abundance-weighted mean normalized affinity:

$$\:{B}_{raw,\:j}=\:\sum\:_{i\in\:j}{f}_{ij}\:(\frac{1}{4}\:\sum\:_{k=1}^{4}{S}_{ik})\:$$

and normalized across oils using a min–max transformation:

$$\eqalign{ {B_{norm,j}} & = \left( {{B_{raw,j}} - {B_{min}}} \right)/ \cr & \left( {{B_{max}} - {B_{mine}}} \right),{B_{norm,j}} \in \left[ {0,1} \right] \cr}$$

Predicted safety was defined as:

$$\eqalign{ & Safet{y_j} = \cr &1 - Essential\_Oil\_Toxicity\_Index/10 \cr}$$

Finally, the IMT for oil (*j*) was defined as:

$$\eqalign{ & IM{T_j} \cr & = 10 \times (\left( {0.55 \times {B_{norm,j}}} \right) \cr & + \left( {0.30 \times OilBreadt\,{h_j}} \right) \cr & + \left( {0.15 \times Safet\,{y_j}} \right)) \cr}$$

The IMT score ranges from 0 to 10, with higher values indicating EOs that exhibit stronger molecular binding, broader neural target engagement, and more favorable predicted safety profiles.

### Chemicals and reagents

German chamomile EO (*Matricaria chamomilla L.*) (Asterales: Asteraceae) was obtained from Bloom Pharmacy (Cairo, Egypt). Commercial freshwater fish feed (AQUA Group™) was obtained from AQUA Group (Cairo, Egypt). Acetone solvent (1 L), Phosphate Buffered Saline (PBS; 10×, pH 7.4, Molecular Biology Grade), formalin solution (10%), ethanol, xylene, and hematoxylin and eosin (H&E) stain were obtained from Piochem Laboratory Chemicals (Giza, Egypt). Mouse monoclonal anti–Caspase-3 antibody (Clone 31A1067, Cat. No. MC0123) was obtained from Medaysis (CA, USA). Horseradish peroxidase (HRP)–conjugated anti-polyvalent secondary antibody and streptavidin reagent were purchased from ScyTek Laboratories (Logan, UT, USA). 3,3′-Diaminobenzidine (DAB) chromogen was obtained from Sigma-Aldrich Sigma-Aldrich. The Amplex™ Red Acetylcholine/Acetylcholinesterase Assay Kit (Cat. No. A12217) was obtained from Thermo Fisher Scientific (Waltham, MA, USA). The Octopamine ELISA Kit (Cat. No. MBS726911) was purchased from MyBioSource (San Diego, CA, USA). The GABA-B Receptor Colorimetric Cell-Based ELISA Kit (Cat. No. EKC1230) was obtained from Boster Biological Technology (Pleasanton, CA, USA).

### Gas chromatography–mass spectrometry (GC–MS) analysis

The chemical composition of the oil was analyzed using a Trace GC-TSQ mass spectrometer (Thermo Scientific, Austin, TX, USA) equipped with a TG–5MS direct capillary column (30 m × 0.25 mm i.d., 0.25 μm film thickness). The oven temperature program was initially set at 50 °C, then increased at a rate of 5 °C/min to 250 °C and held for 2 min. Subsequently, the temperature was raised to a final temperature of 300 °C at a rate of 30 °C/min and held for an additional 2 min. The injector and MS transfer line temperatures were maintained at 270 °C and 260 °C, respectively. Helium was used as the carrier gas at a constant flow rate of 1 mL/min. A solvent delay of 4 min was applied. Diluted samples (1 µl) were injected automatically using an AS1300 autosampler coupled to the GC system, operating in split mode. Electron ionization (EI) mass spectra were acquired at an ionization energy of 70 eV over a mass range of m/z 50–650 in full scan mode. The ion source temperature was set at 200 °C [26]. Compound identification was performed by comparing the obtained mass spectra with those available in the WILEY 09 and NIST 14 mass spectral libraries to ensure accurate and reliable characterization of the EO constituents. Retention indices (RI) were obtained from the NIST Mass Spectral Library and used as supportive identification parameters. Experimental RI values were not determined because a homologous n-alkane series was not analyzed under the same chromatographic conditions.

### Insect and rearing

Third-instar larvae of *Cx. pipiens* were used as an experimental model. Egg rafts were collected from a pond in Tanta Governorate, Egypt, and transported to the animal facility at the Faculty of Science, Tanta University. Hatched larvae were identified to species level based on morphological characteristics following Harbach’s taxonomic key [27]. Larvae were reared on a commercial freshwater fish feed (AQUA Group™) until pupation. Pupae were transferred to plastic cups and maintained in rearing cages (60 × 60 × 60 cm). Following adult emergence, female mosquitoes are blood-fed on a pigeon to stimulate oviposition. The resulting egg rafts were allowed to hatch, and larvae were reared to the third instar, which was used for the subsequent experiments. All specimens were maintained under controlled laboratory conditions (27 ± 2 °C, 55–65% relative humidity, 12:12 h light: dark photoperiod). All rearing and handling procedures complied with the ethical standards of the Research Ethics Committee at the Faculty of Science, Tanta University (Institutional Animal Care and Use Committee SCI-TU-0528).

### Larvicide toxicity

The insecticidal activity of German chamomile EO was evaluated against third-instar *Cx. pipiens* larvae following the protocol described by (Mansour et al., 2025). A stock solution of 2000 ppm was prepared by dissolving 1 ml of pure EO (100% v/v) in 1 ml of acetone to ensure complete solubilization and then adjusting the final volume to 500 ml with distilled water. Subsequent test concentrations (100, 150, 200, 250, 300, 400, and 500 ppm) were prepared by serial dilution from the stock solution using the standard dilution equation (C₁V₁ = C₂V₂). A negative control consisting of distilled water and a positive control containing the same volume of acetone as used in the treated samples were included. For each concentration, 50 ml of the prepared solution was dispensed into plastic cups (100 ml), and ten larvae were added to each cup. Five replicates were conducted for each concentration under the aforementioned laboratory conditions. Larval mortality was recorded after 24 and 48 h of exposure. Mortality data were analyzed using probit analysis to estimate LC₅₀ values and sublethal concentrations [28]. Morphological alterations in treated larvae were examined and documented using an Olympus CX21 light microscope (Olympus Corporation, Tokyo, Japan). Observations were performed using a 4× objective lens and a 10× eyepiece lens, resulting in a total magnification of ×40. Photomicrographs were captured using a smartphone digital camera mounted on the microscope eyepiece.

### Histological and immunohistochemical assays

#### Histological assay

*Cx. pipiens* larvae from both control and treated groups were carefully collected, washed with 0.9% saline solution, and immediately fixed in 10% formalin at 4 °C for 24 h following standard histological procedures described by [29]. The fixed larvae were subsequently dehydrated through a graded ethanol series, air-dried, and embedded in paraffin wax at 60 °C. After cooling the paraffin blocks to 25 °C, sections of 5 μm thickness were prepared using a Leica Microsystems Leica RM2125 RTS microtome. The obtained sections were stained with hematoxylin and eosin (H&E) stain and was examined using a Leitz–Wetzlar photomicroscope (Ernst Leitz GmbH, Wetzlar, Germany). Photomicrographs were captured using a digital camera, and histological alterations in head capsule and internal tissue of treated larvae were compared with those of the control group [30].

#### Immunohistochemical assay

To evaluate apoptosis, immunohistochemical staining was conducted using an anti–cleaved Caspase-3 antibody. Third instar larvae of *Cx. pipiens* (*n* = 5) were fixed in 10% formalin and subsequently processed through a graded ethanol dehydration series. The samples were then incubated overnight at 4 °C with a mouse monoclonal anti–Caspase-3 antibody (1:200 dilution). This was followed by incubation with a horseradish peroxidase (HRP)–conjugated anti-polyvalent secondary antibody for 30 min at room temperature. The sections were then treated with streptavidin for 10 min and developed using 3,3′-diaminobenzidine (DAB) chromogen for 5–10 min. After immunoreaction, the sections were counterstained with hematoxylin, dehydrated through ascending alcohol concentrations (70%, 80%, 90%, and 100%), cleared in xylene for 5 min, and mounted for microscopic examination. Immunostaining was analyzed using a Leitz–Wetzlar photomicroscope (Ernst Leitz GmbH, Wetzlar, Germany) [31].

### Neural responses of *Culex pipiens* third instar larvae treated with the sublethal concentration of *Matricaria chamomilla* essential oil

#### Sample preparation

To evaluate neural responses of *Cx. pipiens* larvae to German chamomile EO, fifty third-instar larvae were exposed to a previously determined sublethal concentration of the oil (LC_40_, 48 h) (184.25 ppm). After 48 h of exposure, surviving larvae were collected and homogenized in phosphate buffer. Homogenates were kept on ice in precooled tubes to maintain sample integrity prior to centrifugation at 4,500 rpm for 5 min at 4 °C. The resulting supernatant was aliquoted into 0.5 ml portions and stored at − 20 °C for subsequent physiological analyses [32]. A parallel control group was maintained under identical conditions. All treatments were performed in five replicates for both control and experimental groups.

#### Acetylcholinesterase activity

AChE activity was measured using the Amplex™ Red Acetylcholine/Acetylcholinesterase Assay Kit (Catalog No. A12217; Thermo Fisher Scientific, USA), according to the manufacturer’s instructions.

#### Octopamine concentration

Octopamine concentration was quantified using a commercial Octopamine ELISA kit (Catalog No. MBS726911; MyBioSource, USA) according to the manufacturer’s instructions.

#### γ-aminobutyric acid concentration

GABA concentration was determined using a colorimetric cell-based ELISA kit (Catalog No. EKC1230; Boster Bio, USA) following the manufacturer’s protocol.

### Statistical analysis

Mortality data were analyzed using two-way analysis of variance (two-way ANOVA) to evaluate the effects of concentration, exposure time, and their interaction on *Cx. pipiens*. Model assumptions were assessed by testing the normality of residuals using the Shapiro–Wilk test and the homogeneity of variances using Levene’s test. When significant differences were detected, Tukey’s Honestly Significant Difference (Tukey HSD) test was applied for post hoc multiple comparisons. For neurotransmitter analysis, differences between treated and control groups were evaluated using an unpaired Student’s t-test. Data visualization was performed using the ggplot2 [33] package in R (version 4.5.2) [34], and statistical significance was considered at *p* < 0.05.

## Results

### Essential oil dataset preparation

Data on EOs and their chemical compositions were systematically retrieved from three specialized databases: the sCentIndb, EOUdb, and AromaDb. The sCentIndb repository provided information on 2,169 EOs comprising 3,082 chemical compounds. The EOUdb contributed 4,116 EOs and 3,481 associated compounds, while AromaDb added 212 EOs and 572 compounds. After merging and harmonizing all datasets, duplicate records and inconsistent compound identifiers were removed. The final integrated dataset comprised 5,183 unique EOs and 5,069 distinct phytochemical compounds, standardized using PubChem CIDs. A detailed list of all EOs, along with their corresponding compounds and relative abundance data, is provided in Additional file 1.

### Predicted toxicity of essential oil constituents

Analysis of EO constituents revealed notable differences in their predicted toxicity profiles. Compounds with low predicted vertebrate toxicity were predominantly fatty acid esters, menthane monoterpenoids, carboxylic acid esters, and unsaturated aliphatic hydrocarbons, and certain sesquiterpenoids. These chemical classes consistently exhibited higher predicted LD₅₀ values, low toxicity class assignments, and minimal alerts for organ- or receptor-specific toxicities, suggesting that EOs enriched in these constituents are likely to be safer including compounds such as cedryl acetate, longiborneol acetate, 3-methylbutyl 4-methylpentanoate, and glyceryl monooleate. In contrast, compounds predicted to have higher toxicity were mainly branched alkanes, straight-chain alkanes, ketones, primary alcohols, and cycloalkanes, and some sesquiterpenoids. EOs containing larger proportions of these compounds may carry greater toxicological risk, as indicated by lower predicted LD_50_ values, less favorable acute toxicity predictions and potential organ- or receptor-mediated effects including compounds such as Warfarin and Calcitriol. These results indicate that the chemical composition of EOs may serve as a useful predictor of their relative safety, providing a rational basis for selecting oils with the lowest predicted toxicity for further experimental evaluation.

### Toxicity distribution and selection of low-toxicity essential oils

**Fig. 1:**
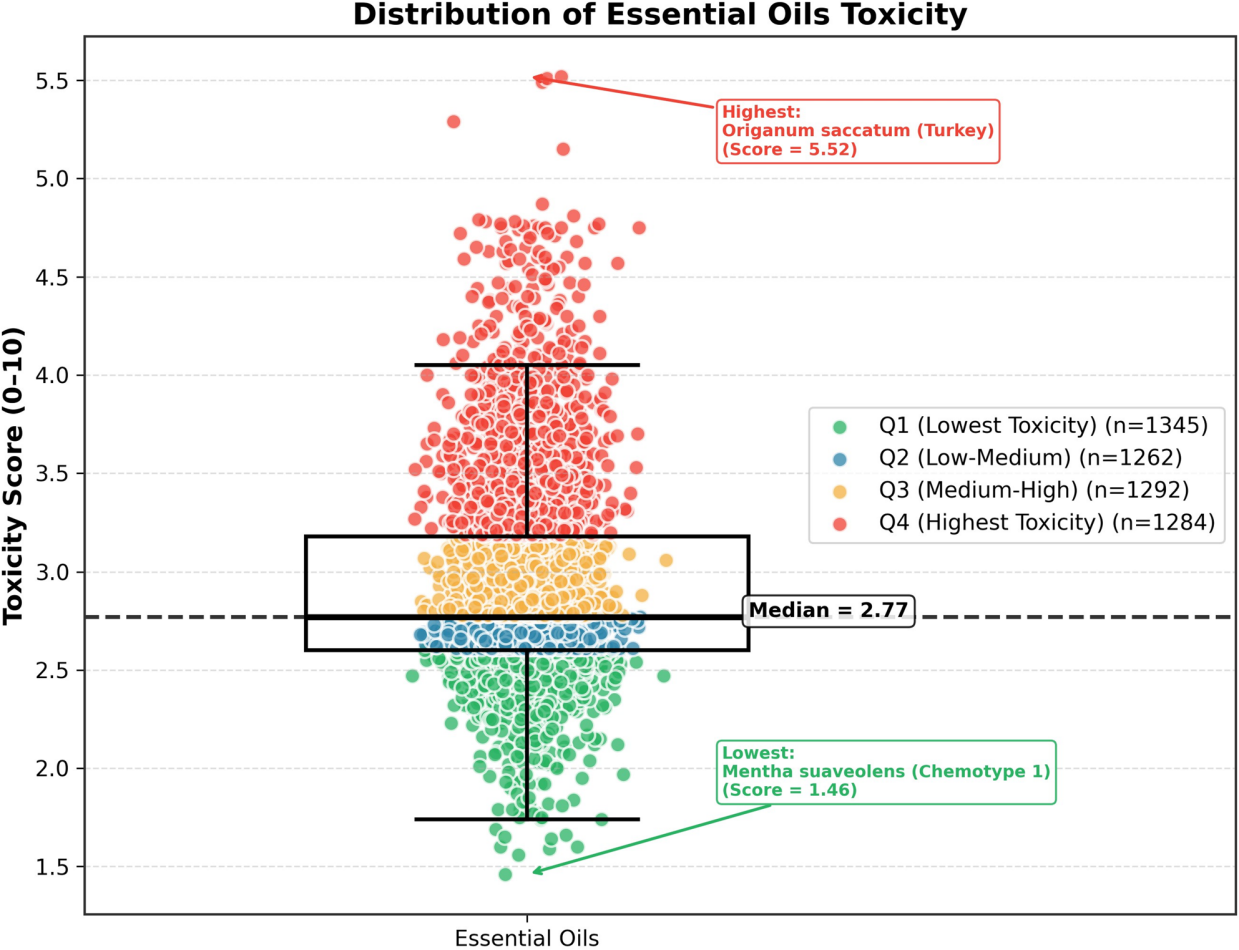
Distribution of predicted toxicity scores for 5,183 essential oils. The boxplot shows the median (2.77), interquartile range, and score range (1.46 to 5.52)

The toxicity scores of 5,183 EOs were calculated using the multi-parameter toxicity model described in Sect.  "Toxicity scoring and oil-level aggregation". The resulting scores ranged from 1.46 to 5.52, with a median value of 2.77. The distribution of toxicity scores across all oils is presented in Fig. 1. EOs with the lowest predicted toxicity were selected for further molecular docking analyses. This filtering resulted in 1,345 oils, representing 2,271 unique phytochemical compounds, which were retained for subsequent target-based screening against mosquito-related molecular targets. Detailed profiles of the selected low-toxicity EOs, including their constituent compounds and final toxicity scores, are provided in Additional file 2.

### Integrate docking and target affinity analysis

**Fig. 2:**
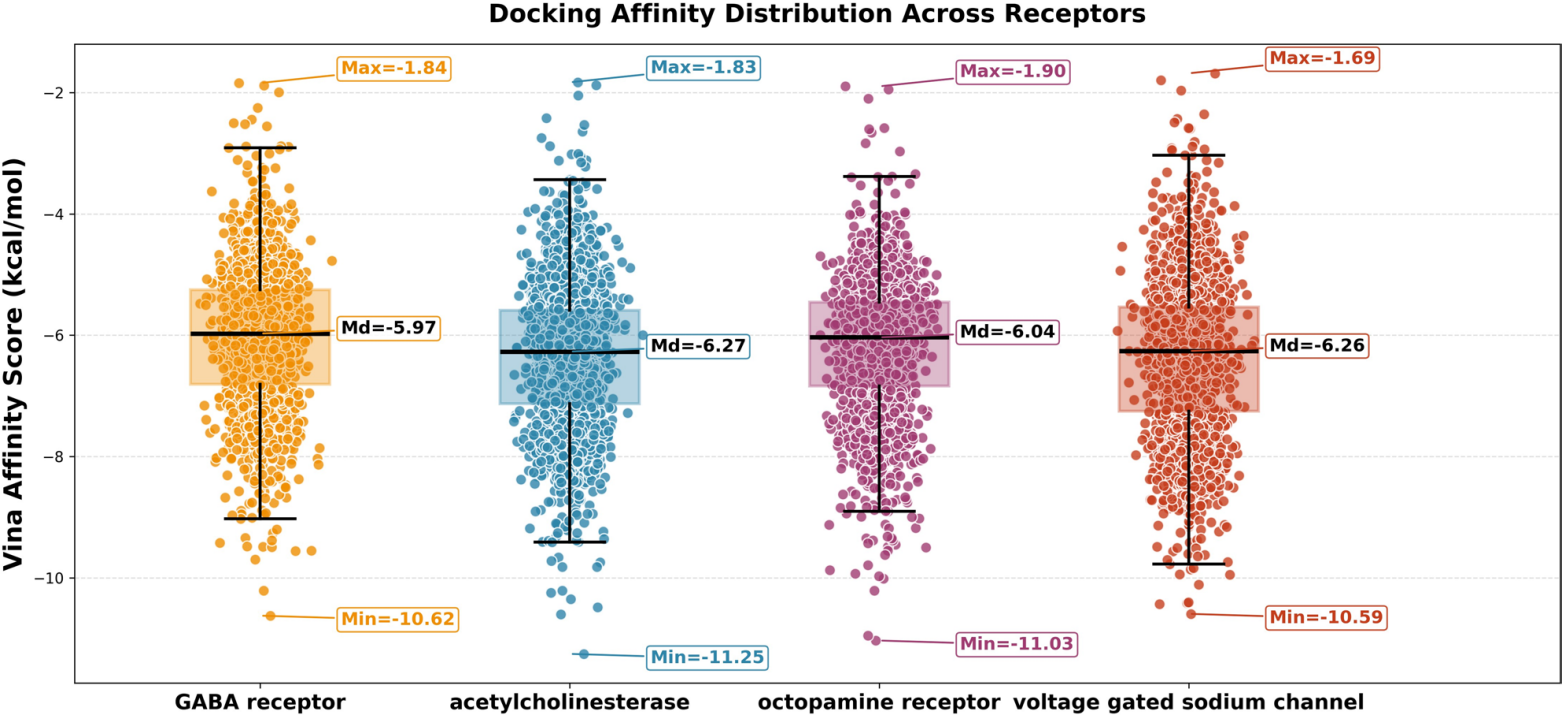
Distribution of predicted binding affinities (kcal.mol^− 1^) for 1345 essential oil–derived compounds across the four neural targets

A total of 2,253 phytochemical compounds were successfully docked against the four mosquito neural protein targets: AChE, VGSC, GABA_R_, and OAR. The reference ligands—chlorpyrifos, indoxacarb, fipronil, and amitraz, respectively—were used to validate the docking protocol. The binding site coordinates for each target, along with docking validation parameters and structural quality metrics, are summarized in Table 1. Binding-pocket prediction using P2Rank also produced high probability scores across all targets, supporting the suitability of the selected active sites for molecular docking. The predicted binding site residue demonstrated high overall confidence, with average pLDDT values ranging from 81.82 to 96.43, indicating reliable structural quality within the analyzed binding regions. Re-docking of the reference ligands through 50 independent docking runs produced low average RMSD values relative to the best-scoring poses, confirming the reproducibility and stability of the docking protocol. Predicted binding affinities for the EO–derived compounds showed moderate variation across targets (Fig. 2). The median affinities were − 6.27 for AChE (range: − 11.25 to − 1.83), − 6.26 for the VGSC (–10.59 to − 1.69), − 6.04 for the OAR (–11.03 to − 1.90), and − 5.97 for the GABA_R_ (–10.62 to − 1.84). The docking results revealed a subset of compounds with strong and consistent binding across the four selected mosquito neural receptors. The highest-performing compound, CID 73,145 (beta-Amyrin), showed an average binding affinity of − 10.46 kcal·mol⁻¹, followed by CID 54,173,984 (-9.95 kcal·mol⁻¹), CID 22,213,932 (–9.90 kcal·mol⁻¹) and 6665 (Dihydrocholesterol) (–9.76 kcal·mol⁻¹). To further highlight the most promising EO-derived compounds, the top 50 docked compounds for each target were compiled and compared. Comparative analysis revealed 31 compounds that were consistently ranked among the top 50 candidates across all four neural targets, indicating broad-spectrum binding potential and possible multi-target mosquitocidal activity. After removal of duplicate entries, a total of 77 unique compounds were identified and included in Additional file 3, together with their PubChem CID, compound name, binding affinities toward each receptor, and EO source. Out of the full set of phytochemical constituents analyzed, approximately 550 compounds were found to display average docking affinities below − 7 kcal·mol⁻¹ across the four neural receptors. These consistently favorable binding energies suggest a broad multi-target interaction potential, supporting the predicted biological activity of high-ranking EOs.

**Table 1:** Docking validation metrics, including reference ligand binding affinity and average RMSD from the best docking pose, predicted active-site coordinates, P2Rank pocket prediction probabilities, and average active-site residue confidence scores (pLDDT) for the mosquito neural protein targets acetylcholinesterase (AChE), γ-aminobutyric acid receptor (GABAR), octopamine receptor (OAR), and voltage-gated sodium channel (VGSC) used in molecular docking analyses

	AChE	GABAR	OAR	VGSC
Reference Ligand	Chlorpyrifos	Fipronil	Amitraz	Indoxacarb
Binding Affinity (kcal/mol)	-7.21	-7.74	-8.94	-9.16
Average RMSD from Best Pose (Å)	0.24	0.08	0.64	1.53
P2Rank Probability	0.97	0.976	0.84	0.933
Center X (Å)	-7.59	27.27	-20.29	0.19
Center Y (Å)	-2.75	6.6	20.15	5.34
Center Z (Å)	2.87	-26.72	6.55	-16.85
Binding Site Residues	A_197, A_198, A_200, A_202, A_212, A_213, A_245, A_246, A_247, A_249, A_250, A_255, A_258, A_326, A_327, A_408, A_409, A_411, A_412, A_413, A_414, A_415, A_416, A_456, A_457, A_460, A_461, A_567, A_568	A_455, A_508, A_511, A_515, A_517, A_518, A_527, A_531, A_534, A_535, A_538, A_539, A_542, A_545, A_549, A_593, A_599, A_624, A_625, A_626, A_627, A_631, A_635, A_638, A_639, A_642, A_643, A_646, A_677, A_680, A_681, A_682, A_684, A_685, A_688, A_692, A_695, A_698, A_699, A_702, A_705, A_706, A_709, A_710, A_713	A_254, A_257, A_258, A_261, A_262, A_273, A_277, A_347, A_348, A_349, A_350, A_352, A_663, A_666, A_667, A_670, A_678, A_680, A_683, A_684, A_687, A_688, A_691	A_1045, A_1078, A_1079, A_1081, A_1082, A_1083, A_1085, A_1086, A_1087, A_1089, A_485, A_486, A_493, A_496, A_497, A_500, A_539, A_543, A_546, A_547, A_577, A_580, A_581, A_583, A_584
Average pLDDT	96.43	85.96	84.83	81.82

### Evaluation of essential oil potency using the IMT index

The integrated potency assessment of the 1,345 low-toxicity EOs revealed substantial variation in their predicted biological efficacy, with IMT values ranging from 1.11 to 7.73. Oils with higher IMT values demonstrated a favorable balance between predicted binding strength, neural target coverage, and overall safety. The top-performing oils are summarized in Table 2, while the complete ranking of all assessed oils is provided in Additional file 4. To further refine the selection of experimentally relevant candidates, EOs with IMT values greater than 5 were examined, yielding a subset of 51 high-potency oils. Within this subset, several botanical species were represented by multiple chemotypic variants. Notably, Chamomile, germen appeared in eight distinct chemotypes, including Chamomile, german (Germany) 1, Chamomile, german (Italy) 1, Chamomile, german (Italy) 2, Chamomile, german (Hungary), Chamomile, german (Brazil), Chamomile, german (Argentina), Chamomile, german (India) 1, and Chamomile, german (Egypt). The recurrent appearance of German chamomile EO across different sources indicated both broader chemical consistency and wider natural availability. Consequently, Chamomile (Egypt) was selected as a representative and readily accessible candidate for subsequent laboratory-based validation and further experimental investigation.

**Table 2:** Top ten essential oils ranked by the Integrated Mosquito Target Index (IMT), with corresponding dataset sources and predicted toxicity values

Essential Oil Name	Scientific Name	IMT	Predicted Toxicity	Source
Cedarwood Texas 2	*Juniperus mexicana*	7.74	2.57	Eoudb
Chamomile, German (Germany) 1	*Matricaria chamomilla*	7.53	2.32	Eoudb
*Araucaria cunninghamii* (leaf, leaf)	*Araucaria cunninghamii*	7.57	2.57	sCentlndb
*Cupressus funebris*	*Cupressus funebris*	7.4	2.35	Eoudb
Chamomile, German (Italy) 2	*Matricaria chamomilla*	7.19	2.03	Eoudb
Chamomile, German (Italy) 1	*Matricaria chamomilla*	7.19	2.23	Eoudb
Chamomile, German (Hungary)	*Matricaria chamomilla*	7.03	2.32	Eoudb
*Eugenia uniflora* leaf (Brazil) 1	*Eugenia uniflora*	6.97	2.26	Eoudb
*Aquilaria sinensis*	*Aquilaria sinensis*	6.85	2.11	Aromadb

### Comparative chemical composition analysis

**Fig. 3:**
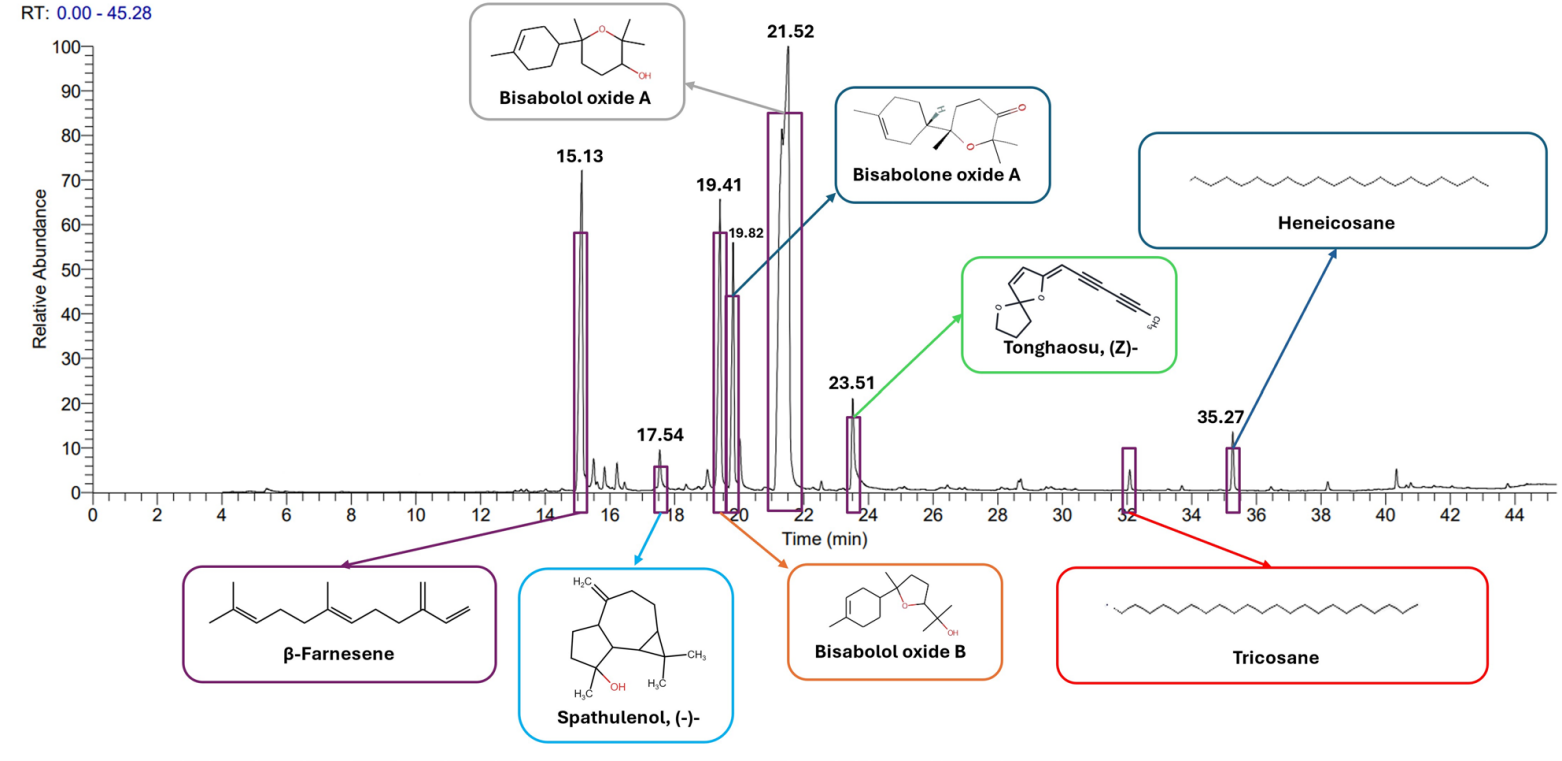
GC–MS chromatogram of the experimentally Egyptian *Matricaria chamomilla* essential oil showing the relative abundance of identified compounds

A comparative analysis was performed between the eight high-ranking German chamomile chemotypes and the experimentally analyzed German chamomile (Egypt) EO using GC–MS. The major constituents identified across the eight chemotypes were mainly bisabolol oxides (A and B) and β-farnesene, while minor components included chamazulene, β-caryophyllene, germacrene D, tonghaosu, p-cymene, and spathulenol. GC–MS analysis of the experimentally tested oil revealed a closely related chemical profile (Fig. 3; Table 3), with bisabolol oxide A as the dominant compound, followed by β-farnesene, bisabolol oxide B, bisabolone oxide A, tonghaosu, spathulenol, and germacrene D. Trace amounts of T-cadinol, caryophyllene oxide, and p-cymene were also detected. The chemical composition of the analyzed oil largely resembles the characteristic bisabolol-rich chemotype observed in the eight high-ranking variants, due to predominance of oxygenated sesquiterpenes and sesquiterpene hydrocarbons. The high abundance of these derivatives supports the suitability of this oil for subsequent biological validation.

**Table 3:** Chemical composition of the experimentally Egyptian *Matricaria chamomilla* essential oil identified by GC–MS analysis

RT	Area %	Compound Name	CID	MW	MF	RI	Reference
21.52	28.99	Bisabolol oxide A	13,092,559	238.37	C₁₅H₂₆O₂	1748	[75, 76]
21.31	17.97	(3 S,6 S)-2,2,6-trimethyl-6-[(1R)-4-methylcyclohex-3-en-1-yl]oxan-3-ol (Bisabolol oxide A Stereoisomer)	13,092,558	238.37	C₁₅H₂₆O₂	1734	[77]
15.13	13.21	beta-Farnesene	5,281,517	204.35	C₁₅H₂₄	1448	[76]
19.41	9.85	Bisabolol oxide B	117,301	238.37	C₁₅H₂₆O₂	1644	[75]
19.82	7.16	Bisabolone oxide A	91,700,388	236.35	C₁₅H₂₄O₂	1670	[75, 76]
23.51	4.45	Tonghaosu, (Z)-	5,352,494	200.23	C₁₃H₁₂O₂	1634	[78]
20.02	1.67	alpha-Bisabolol	10,586	222.37	C₁₅H₂₆O	1683	[76]
35.27	1.42	Heneicosane	12,403	296.6	C₂₁H₄₄	2100	
17.54	1.19	Spathulenol, (-)-	13,854,255	220.35	C₁₅H₂₄O	1569	[76]
15.5	1.02	Germacrene D	5,317,570	204.35	C₁₅H₂₄	1480	[75, 76]
19.01	0.8	T-Cadinol	160,799	222.37	C₁₅H₂₆O	1628	
16.21	0.79	alpha-Farnesene	5,281,516	204.35	C₁₅H₂₄	1499	[78]
15.83	0.77	Gamma-elemene, (-)-	10,583	204.35	C₁₅H₂₄	1425	[75]
40.33	0.58	(6E,10E,14E,18E)-2,6,10,15,19,23-Hexamethyl-1,6,10,14,18,22-tetracosahexaen-3-ol	5,366,014	426.7	C₃₀H₅₀O	3058	
32.08	0.48	TRICOSANE	12,534	324.6	C₂₃H₄₈	2300	
40.77	0.32	Geranyllinalool	5,365,872	290.5	C₂₀H₃₄O	2020	
43.76	0.31	5alpha-Cholestan-3beta-ol, 2-methylene-	177,843,078	400.7	C₂₈H₄₈O	3115	
16.45	0.3	delta-Cadinene	441,005	204.35	C₁₅H₂₄	1514	
26.43	0.27	Ethyl palmitate	12,366	284.5	C₁₈H₃₆O₂	1968	[78]
38.2	0.26	Heptacosane	11,636	380.7	C₂₇H₅₆	2700	
29.49	0.25	Oleic Acid	445,639	282.5	C₁₈H₃₄O₂	2113	[78]
14.01	0.19	(-)-Caryophyllene	5,281,515	204.35	C₁₅H₂₄	1424	[74]
29.63	0.18	Ethyl Oleate	5,363,269	310.5	C₂₀H₃₈O₂	2171	
20.55	0.17	Caryophyllene oxide	1,742,210	220.35	C₁₅H₂₄O	1576	[75, 79]
18.74	0.15	Farnesene epoxide, E-	5,362,910	220.35	C₁₅H₂₄O	1624	
4.97	0.06	P-Cymene	7463	134.22	C₁₀H₁₄	1011	[75]
7.87	0.02	Artemisyl acetate	524,254	196.29	C₁₂H₂₀O₂	1152	[75]

RT (min): Retention time; Area %: Peak area percentage; MW: Molecular Weight; MF: Molecular Formula; RI: Retention Index; Reference: Literature sources used for compound identification

### Effect of major constituents on mosquito neural targets

**Fig. 4:**
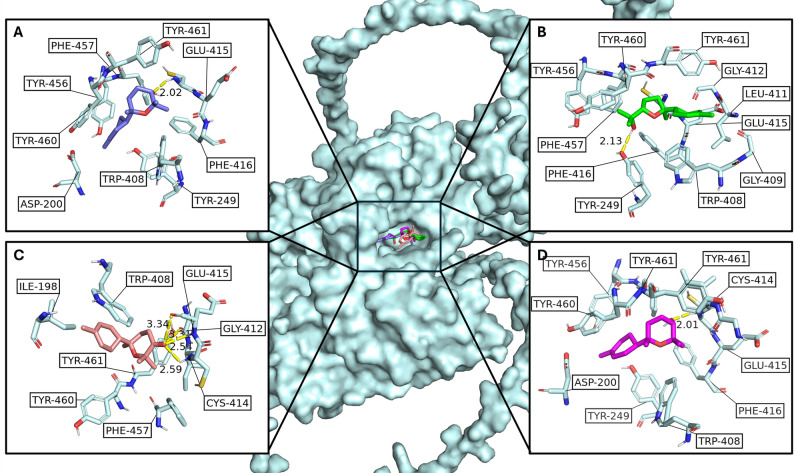
Docking binding poses of the four major bisabolol derivatives — **A**: Bisabolol oxide A (− 7.69 kcal/mol), **B**: Bisabolol oxide B (− 7.92 kcal/mol), **C**: Bisabolone oxide A (− 7.97 kcal/mol), **D**: Bisabolol oxide A stereoisomer (− 7.69 kcal/mol) — with acetylcholinesterase. Yellow dashed lines indicate hydrogen bonds

**Fig. 5:**
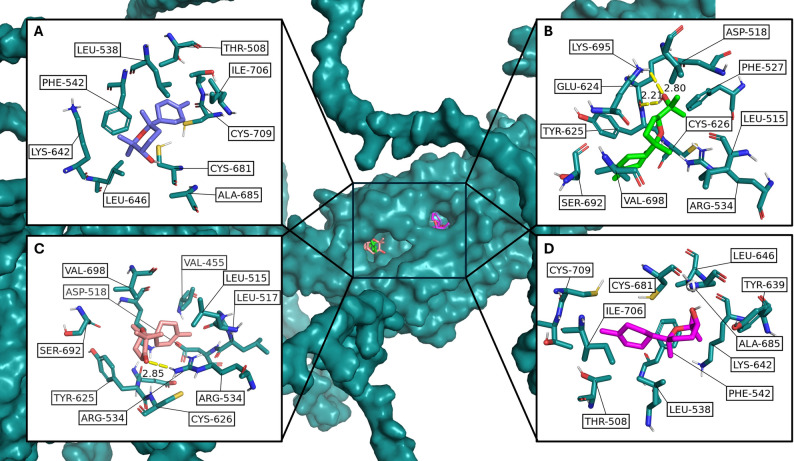
Docking binding poses of the four major bisabolol derivatives — **A**: Bisabolol oxide A (− 7.62 kcal/mol), **B**: Bisabolol oxide B (− 7.46 kcal/mol), **C**: Bisabolone oxide A (− 7.14 kcal/mol), **D**: Bisabolol oxide A stereoisomer (− 7.62 kcal/mol) — with the GABA receptor. Yellow dashed lines indicate hydrogen bonds

**Fig. 6:**
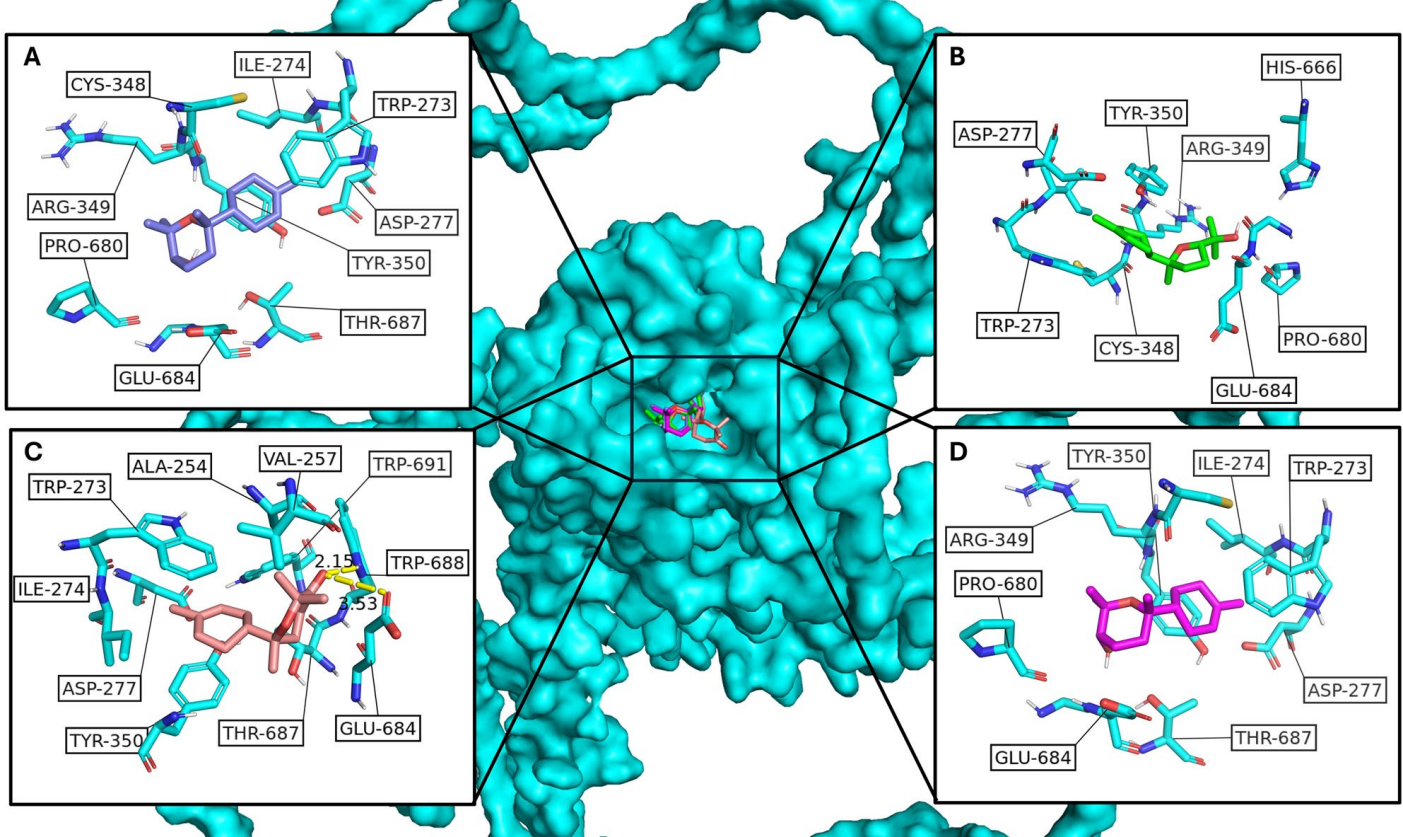
Docking binding poses of the four major bisabolol derivatives — **A**: Bisabolol oxide A (− 7.87 kcal/mol), **B**: Bisabolol oxide B (− 7.68 kcal/mol), **C**: Bisabolone oxide A (− 8.98 kcal/mol), **D**: Bisabolol oxide A stereoisomer (− 7.87 kcal/mol) — with the octopamine receptor. Yellow dashed lines indicate hydrogen bonds

**Fig. 7:**
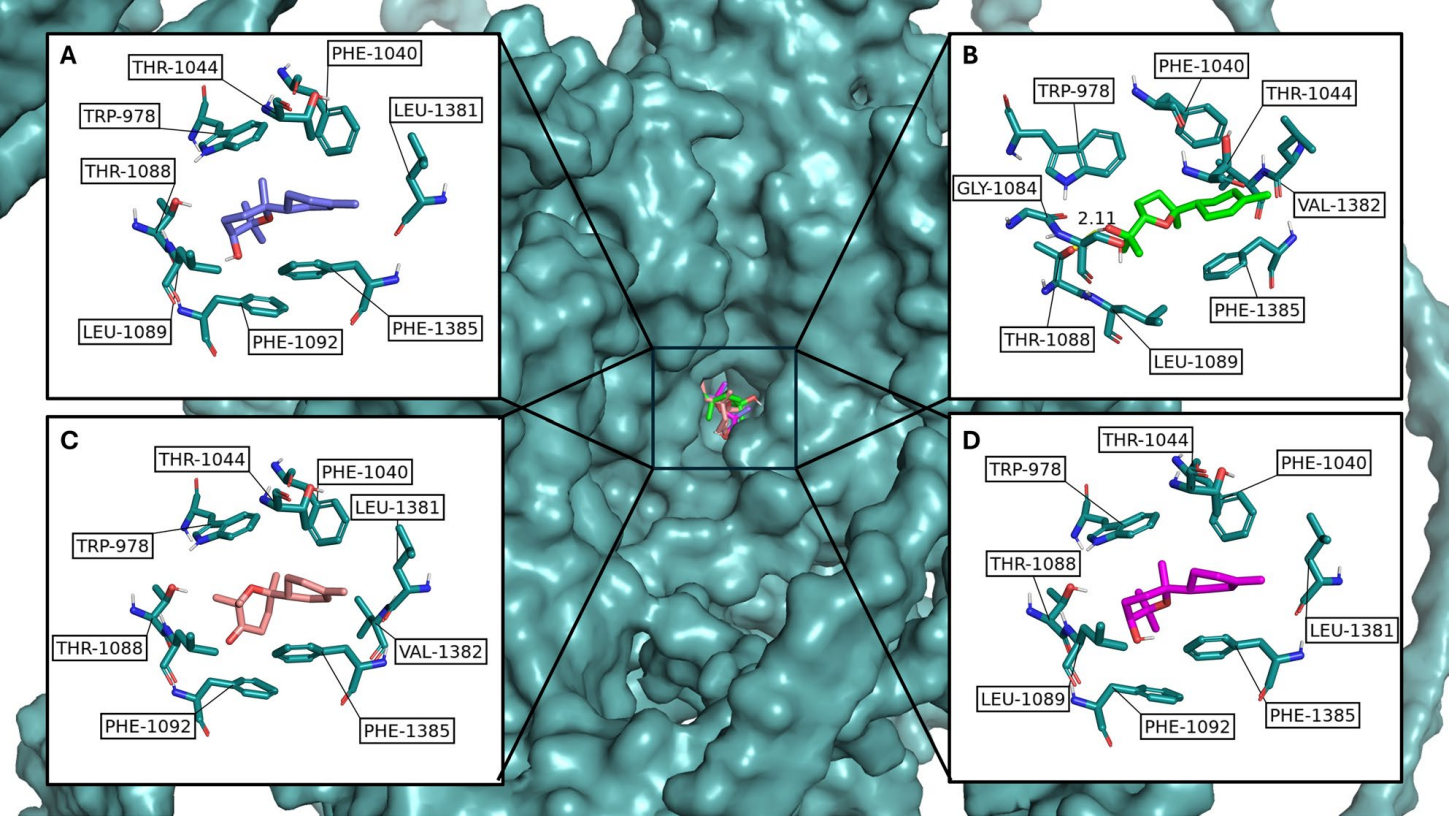
Docking binding poses of the four major bisabomol) derivatives — **A**: Bisabolol oxide A (− 8.44 kcal/mol), **B**: Bisabolol oxide B (− 8.46 kcal/mol), **C**: Bisabolone oxide A (− 8.62 kcal/mol), **D**: Bisabolol oxide A stereoisomer (− 8.46 kcal/mol) — with the voltage-gated sodium channel Yellow dashed lines indicate hydrogen bonds

To further elucidate the molecular basis of the predicted bioactivity, the major constituents identified by GC–MS were docked individually against the four selected mosquito neural targets. The docking scores are summarized in Table 4, while Docking binding interaction diagrams are shown in Figs. 4, 5, 6 and 7. Among the tested compounds, the oxygenated sesquiterpenes, particularly Bisabolone oxide A and Bisabolol oxide A, exhibited the most favorable binding affinities across all targets. Bisabolone oxide A demonstrated the strongest affinity toward the OAR and favorable interactions with the VGSC and AChE. Similarly, Bisabolol oxide A and its stereoisomer showed strong binding to the VGSC and GABA_R_. Bisabolol oxide B also exhibited favorable binding, particularly toward AChE and VGSC. In contrast, hydrocarbon sesquiterpenes such as β-Farnesene and Germacrene D displayed comparatively weaker interactions across all targets, with binding energies generally above − 6.5 kcal/mol. Tonghaosu (Z) showed moderate activity, whereas Spathulenol and Germacrene D demonstrated the lowest affinities. Notably, the compounds with the favorable binding affinities correspond to those present at higher relative abundances in the oil (e.g., Bisabolol oxide A and its stereoisomer), suggesting a positive relationship between chemical composition and predicted multi-target activity. These findings indicate that the biological activity of the oil is likely driven primarily by oxygenated bisabolol derivatives, which exhibit multi-target binding profiles across key mosquito neural proteins.

**Table 4:** Molecular docking binding affinities (kcal/mol) of the major constituents identified by GC–MS against four mosquito neural targets: acetylcholinesterase (AChE), GABA receptor (GABA_R_), octopamine receptor (OAR), and voltage-gated sodium channel (VGSC), and relative abundance (Area %) of each compound in the experimentally Egyptian *Matricaria chamomilla* essential oil

Compound	AChE	GABA_*R*_	OAR	VGSC	area%
Bisabolone oxide A	-7.97	-7.14	-8.98	-8.62	7.16
Bisabolol oxide A	-7.69	-7.62	-7.87	-8.44	28.99
Bisabolol oxide A stereoisomer	-7.69	-7.62	-7.87	-8.46	17.97
Bisabolol oxide B	-7.92	-7.46	-7.68	-8.46	9.85
alpha-Bisabolol	-6.89	-6.92	-7	-7.85	1.67
Tonghaosu, (Z)-	-6.21	-6.14	-6.23	-6.65	4.45
Beta-Farnesene	-6.15	-6.44	-6.36	-6.24	13.21
Spathulenol, (-)-	-6	-5.34	-5.69	-5.64	1.19
Germacrene D	-5.31	-4.94	-4.59	-5.22	1.02

### Larvicidal toxicity assay

**Fig. 8:**
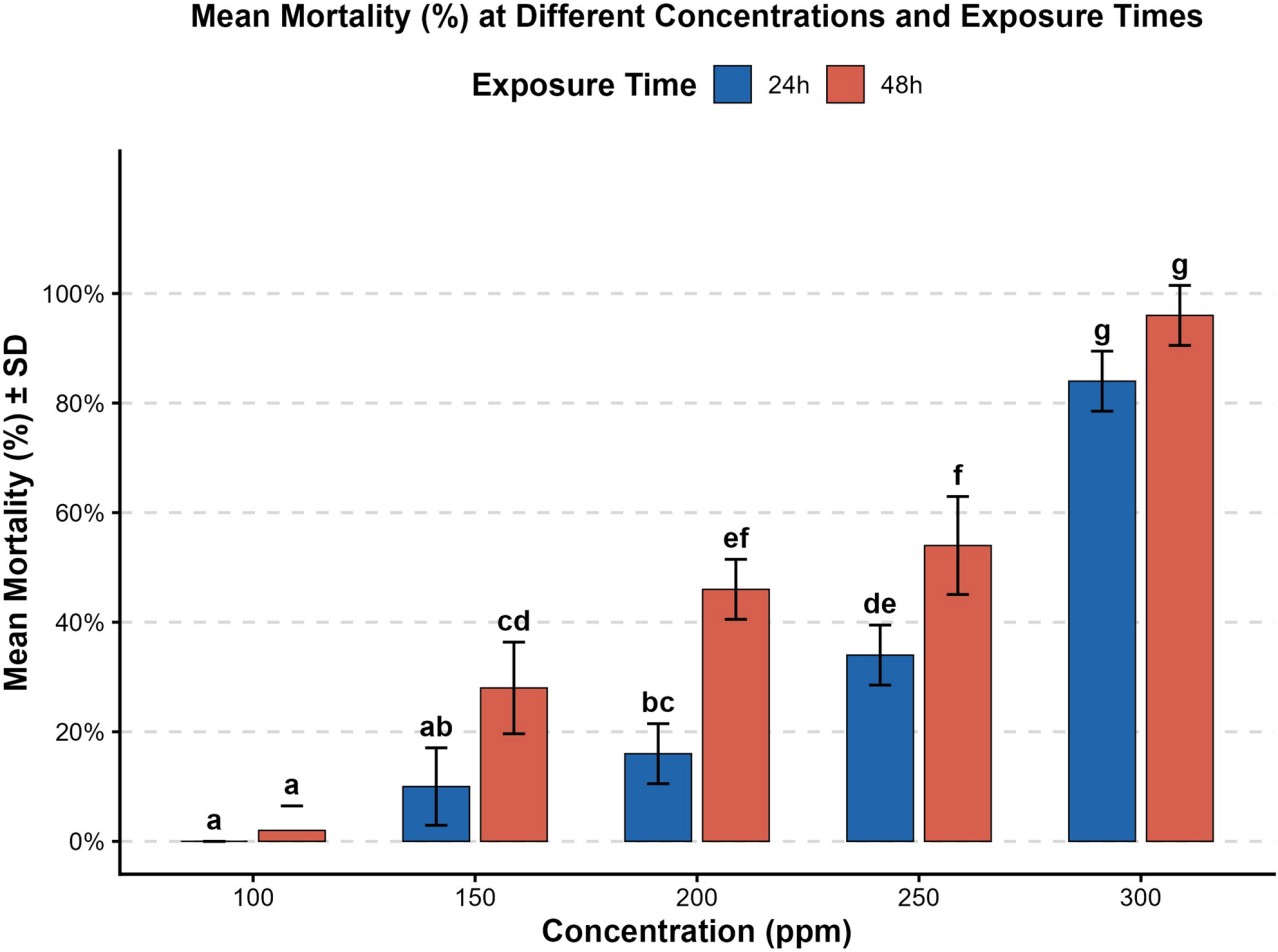
Mortality rates (mean % ± SD) of *Culex pipiens* larvae recorded at 24 and 48 h post exposure to various concentrations of Egyptian *Matricaria chamomilla* essential oil. Bars sharing the different letters indicate a statistically significant difference at (*P* < 0.05)

As shown in Fig. 8, the Egyptian German chamomile EO had a significant effect on the *Cx. pipiens* larval mortality. Two-way ANOVA revealed highly significant effects of concentration (F = 305.0, *P* < 0.001), exposure duration (F = 90.87, *P* < 0.001), and interaction between both factors (F = 7.22, *P* = 0.001). At 24 h post-treatment, mortality increased gradually with increasing concentration, with relatively low mortality at 100–150 ppm and a sharp rise at concentrations ≥ 250 ppm. Complete mortality was observed at 400 and 500 ppm. Extending the exposure period to 48 h further enhanced larvicidal activity, especially at lower concentrations (100–250 ppm), whereas near-complete mortality at higher concentrations (300–500 ppm) was already achieved at 24 h.

**Fig. 9:**
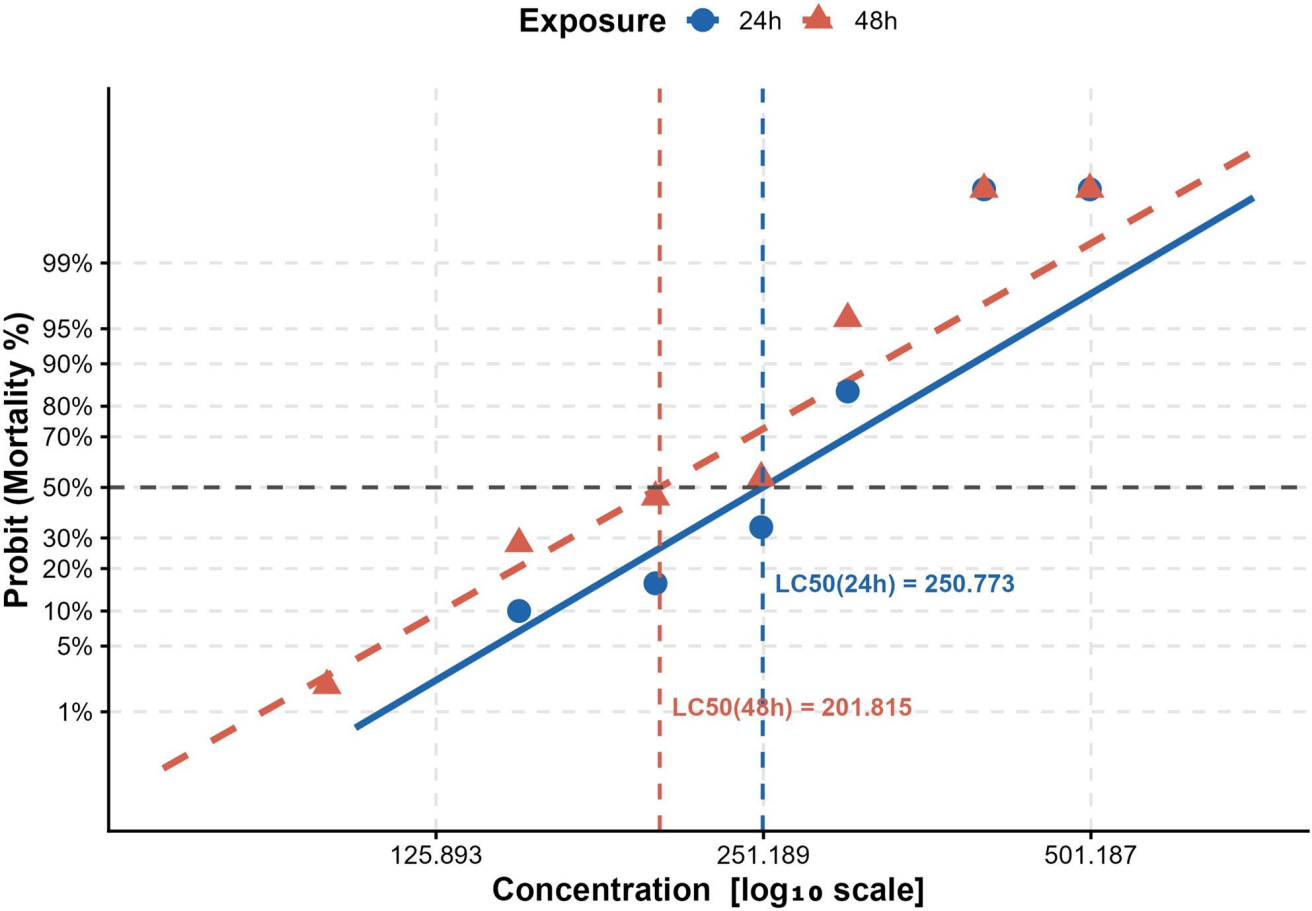
Dose–response curves showing larval mortality (%) of *Culex pipiens* exposed to Egyptian *Matricaria chamomilla* essential oil. Probit analysis was used to estimate LC₅₀ values (Concentration required to induce mortality in 50% of insects) after 24 and 48 h of exposure

Dose–response relationships are illustrated in Fig. 9; Table 5. The fitted models showed good agreement with the observed data for both exposure periods, with non-significant chi-square goodness-of-fit values indicating adequate model performance. Toxicity increased with exposure duration, as reflected by lower lethal concentration estimates at 48 h. The LC₅₀ decreased from 250.77 ppm at 24 h to 201.82 ppm at 48 h, while the LC₉₀ values decreased from 390.06 ppm to 319.92 ppm, respectively.

**Table 5:** Lethal concentrations (LC_50_ and LC_90_) against *Culex pipiens* third instar larvae after 24 and 48 h exposure to different concentrations of Egyptian *Matricaria chamomilla* essential oil

Time(h)	LC_50_ (95% CI)	LC_90_ (95% CI)	Regression equation	Chi² *p*-value
24	250.773	390.056	Y = 7.084x-11.987	0.116
	(220.191-285.601)	(342.490-444.229)		
48	201.815	319.921	Y = 6.988x-11.072	0.229
	(176.160-231.207)	(279.252-366.512)		

### Behavioural and pathological effects of *Matricaria chamomilla* essential oil on *Culex pipiens* larvae

**Fig. 10:**
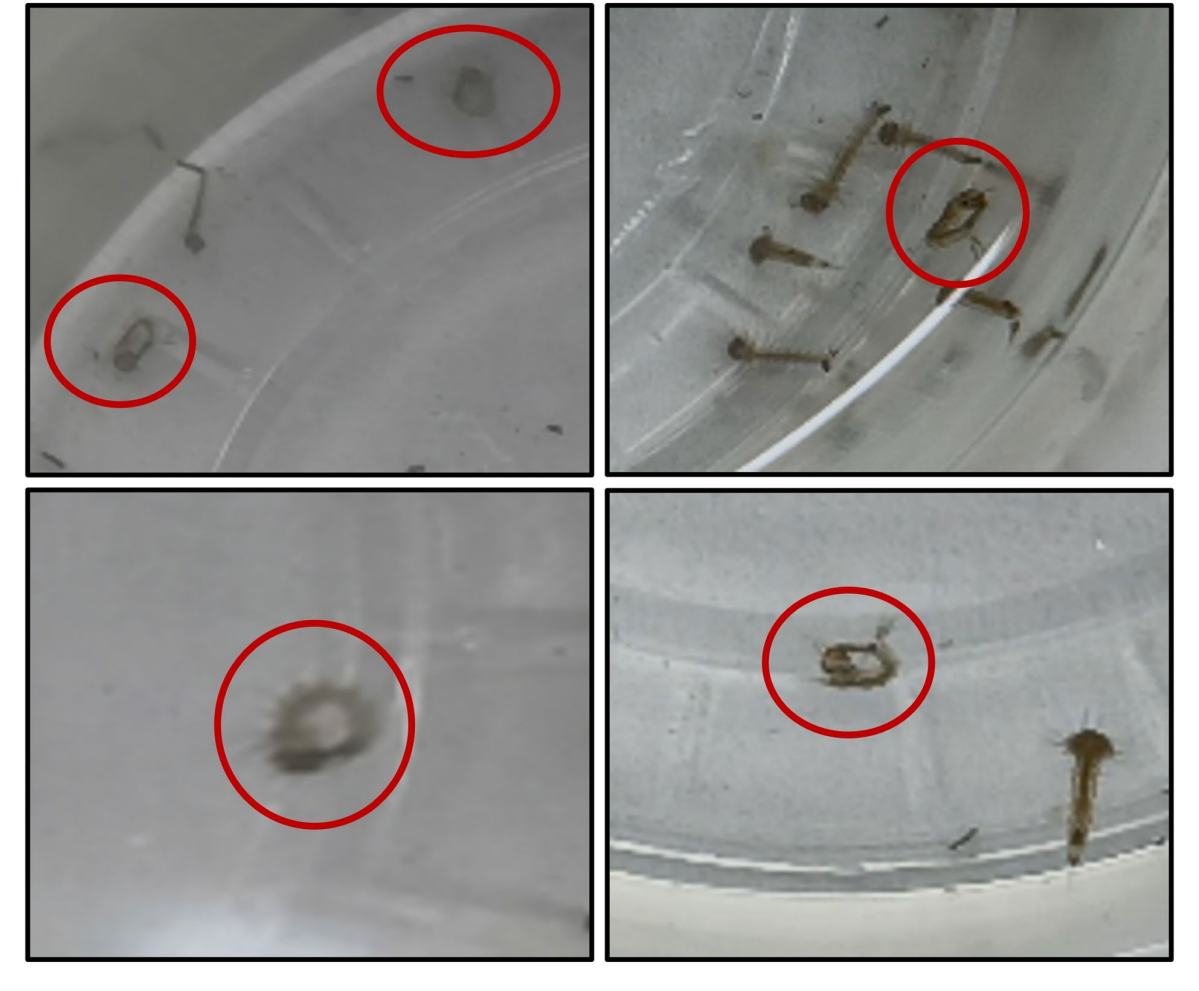
Photomicrographs showing behavioural alterations in *Culex pipiens* larvae following exposure to the sublethal concentrations (LC_40_, 48 h) of Egyptian *Matricaria chamomilla* essential oil. Red circles highlight self-directed biting behaviour targeting the anal papillae

**Fig. 11:**
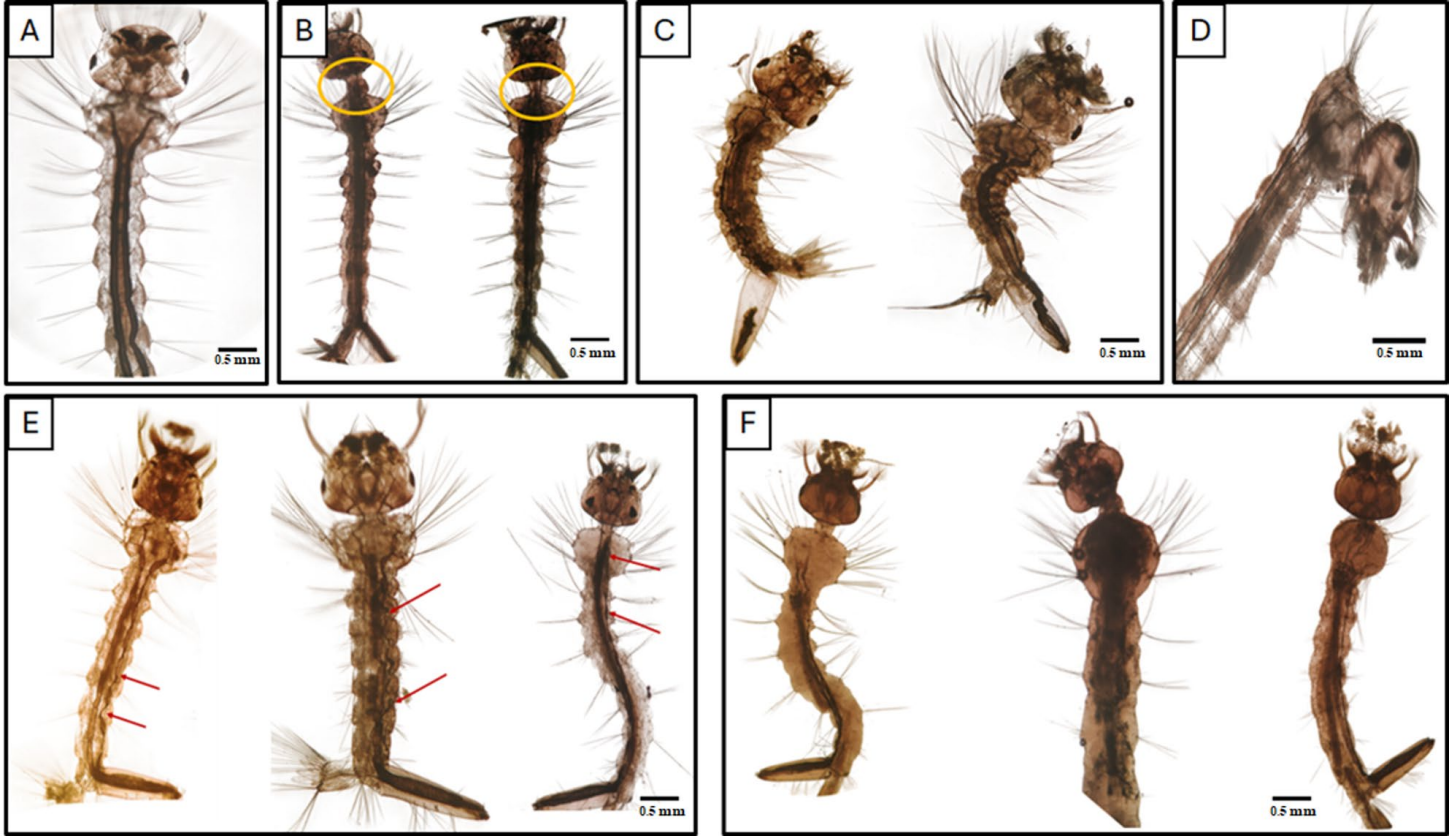
Photomicrographs showing pathological alterations in *Culex pipiens* larvae following exposure to Egyptian *Matricaria chamomilla* essential oil. (**A**) Control larvae exhibiting normal morphological features. (**B**–**F**) Treated larvae showing multiple pathological alterations, including (**B**) increased separation between the head and thorax, (**C**) disruption of the siphon tubular structure, (**D**) partial separation of the head capsule, (**E**) tracheal deformation, and (**F**) internal tissue lysis. Yellow circles indicate increased separation between the head and thorax, while red arrows indicate tracheal deformation. Images were captured using an Olympus CX21 light microscope (Olympus Corporation, Tokyo, Japan) at ×40 magnification

*Cx. pipiens* larvae exposed to the Egyptian German chamomile EO exhibited pronounced behavioural and morphological disturbances compared to the control. Behaviourally, treated larvae displayed hyperactivity, abnormal aggressive movements, and impaired coordination, including repeated twisting of the body and self-directed biting, often targeting the anal papillae (Fig. 10). Morphologically, the control larvae exhibited normal structural features with no observable abnormalities (Fig. 11A). In contrast, the treated larvae showed marked abnormalities, such as an increased separation between the head and thorax, resulting in an extended head–thorax junction (Fig. 11B), pronounced deformities of the siphon with disruption and fragmentation of its tubular structure (Fig. 11C), partial separation of the head capsule (Fig. 11D), deformation of the tracheal system (Fig. 11E), and extensive internal tissue lysis (Fig. 11F), indicating severe structural damage to larval body organization.

### Histopathological alterations and caspase-3–mediated apoptosis in *Culex pipiens* larvae Induced by *Matricaria chamomilla* essential oil

**Fig. 12:**
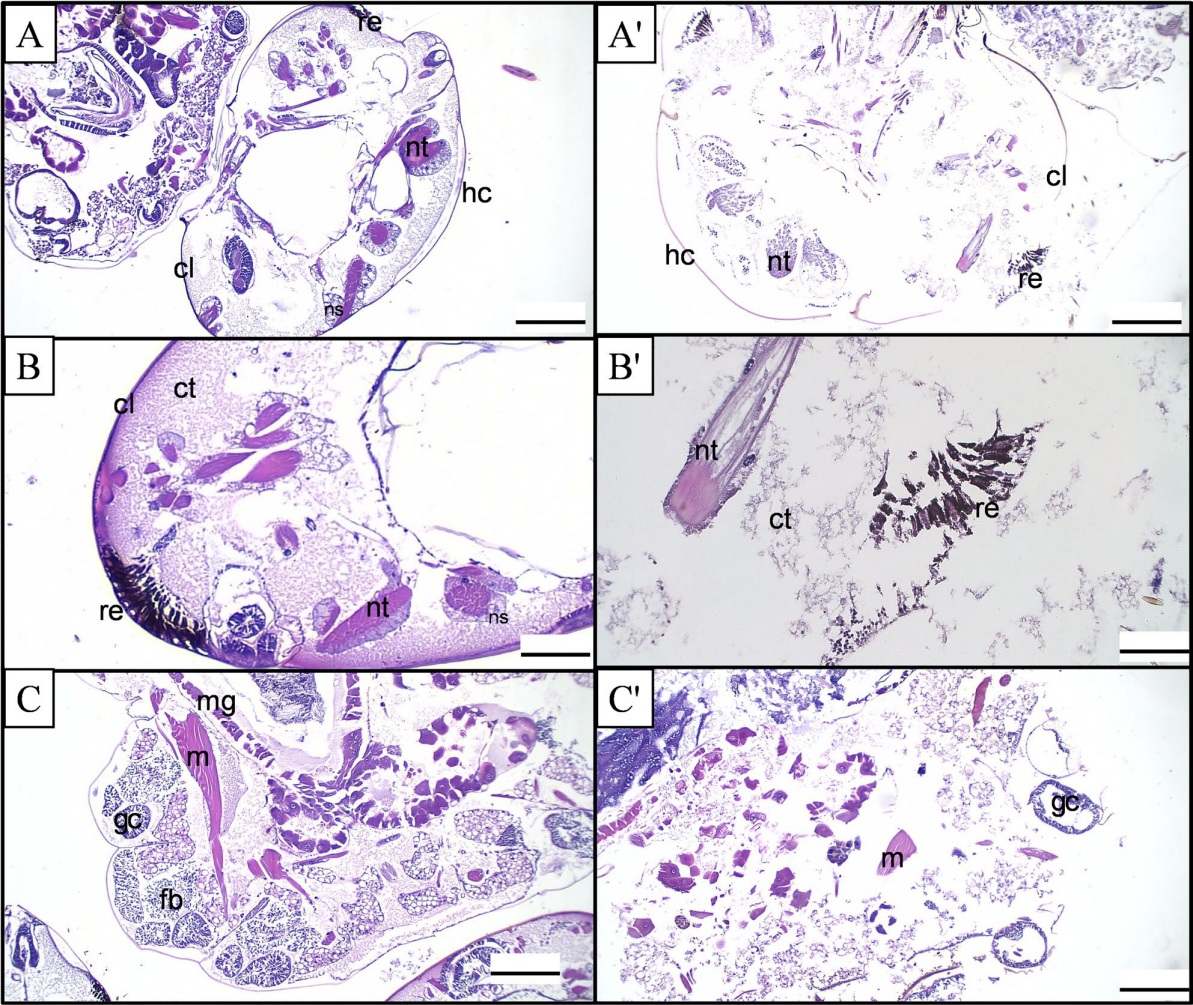
Histological architecture of the head capsule and internal tissues of *Culex pipiens* larvae stained with Hematoxylin and Eosin, in control (**A**–**C**) and treated groups (**A′–C′**), demonstrating the effect of *Matricaria chamomilla* essential oil after 48 h exposure to LC40. The head capsule (hc) consists of cuticular layer (cl), retina (re), neural tissues (nt), neurosecretory cells (ns), and connective tissue (ct). The internal tissues include midgut (mg), gastric caeca (gc), fat body (fb), and muscle (m). scale bar = 100 μm

**Fig. 13:**
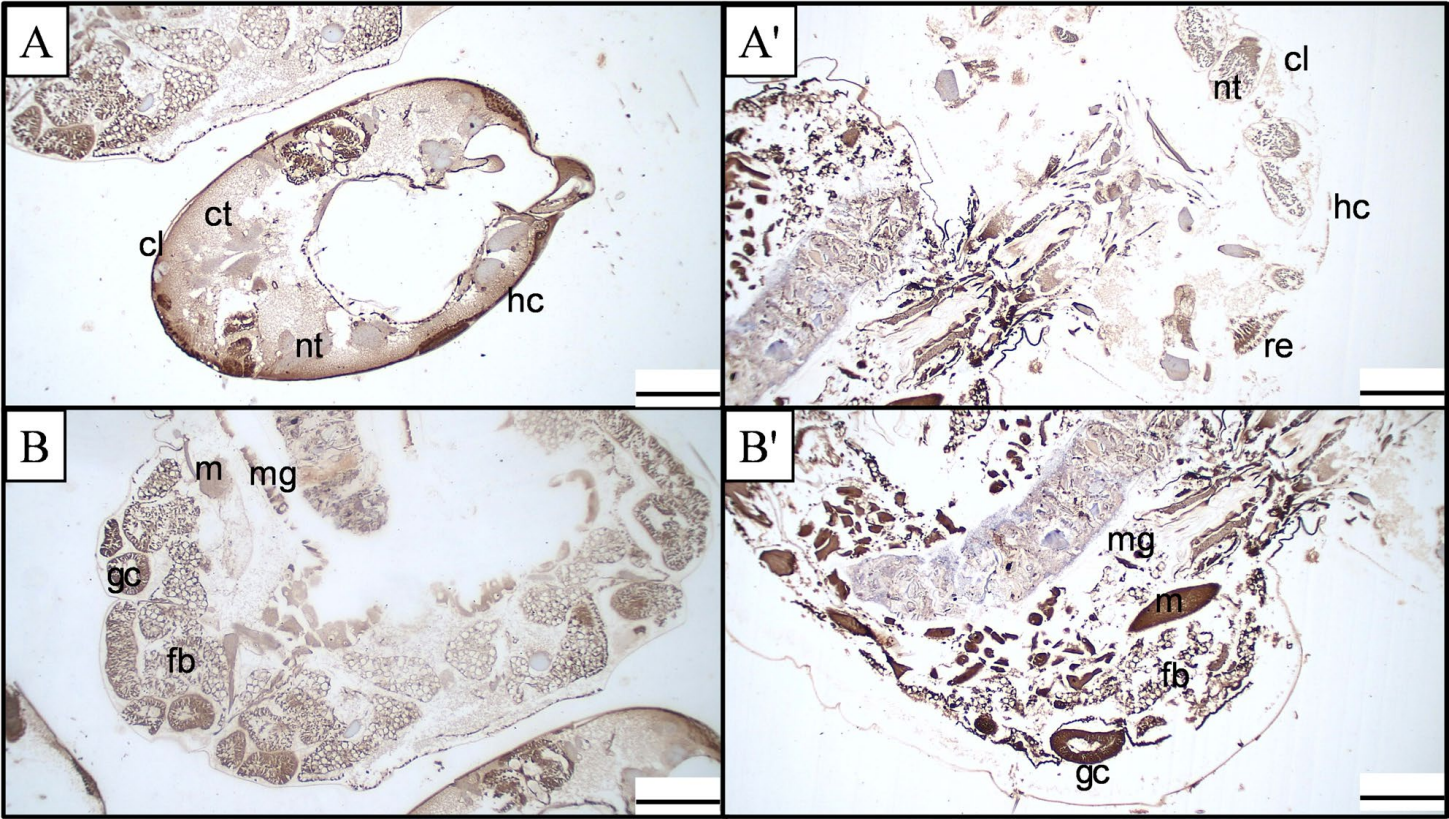
Immunohistochemical analysis of the head capsule and internal tissues of third instar larvae of *Culex pipiens* stained with anti-caspase-3 antibody. (**A**, **B**) Control group; (**A′, B′**) treated group, demonstrating the effect of *Matricaria chamomilla* essential oil after 48 h exposure to LC_40_. The head capsule (hc) consists of cuticular layer (cl), retina (re), neural tissues (nt), neurosecretory cells (ns), and connective tissue (ct). The internal tissues include midgut (mg), gastric caeca (gc), fat body (fb), and muscle (m). scale bar = 100 μm

Hematoxylin and Eosin staining of control larvae showed a normal and well-organized histological architecture of the head capsule (Fig. 12A, B). The cuticular layer appeared intact and clearly defined, while the retina exhibited a compact and regular arrangement. The neural tissues were well preserved with normal structural integrity, and no histopathological alterations were observed. At the level of internal organs (Fig. 12C), the tissues also displayed normal organization and clear differentiation of the midgut, gastric caeca, fat body tissue, and muscles. In contrast, the treated group exhibited severe and extensive histopathological damage, characterized by complete destruction of tissue architecture. Marked disintegration of the retina and neural tissues was observed, along with severe disruption and loosening of the connective tissue (Fig. 12A’, B’). At the level of internal organs, complete degeneration of the digestive tract was evident, with loss of normal structure in the midgut, gastric caeca, and muscle layers, resulting in total loss of tissue differentiation (Fig. 12C’). Immunohistochemical analysis (anti–Caspase-3) revealed a strong positive brown immunoreactivity in the treated larvae compared with the control. The control group showed weak or minimal staining (Fig. 13A, B), whereas in the treated group, the brown signal was markedly intensified and predominantly localized in the remaining detectable and degenerating tissues. This included neural tissues, muscle, gastric caeca, and areas showing necrosis, indicating that the residual structures exhibited enhanced Caspase-3 expression associated with extensive cellular damage and apoptosis (Fig. 13A’, B’).

### Physiological responses of *Culex pipiens* larvae following exposure to a sublethal concentration of *Matricaria chamomilla* essential oil

**Fig. 14:**
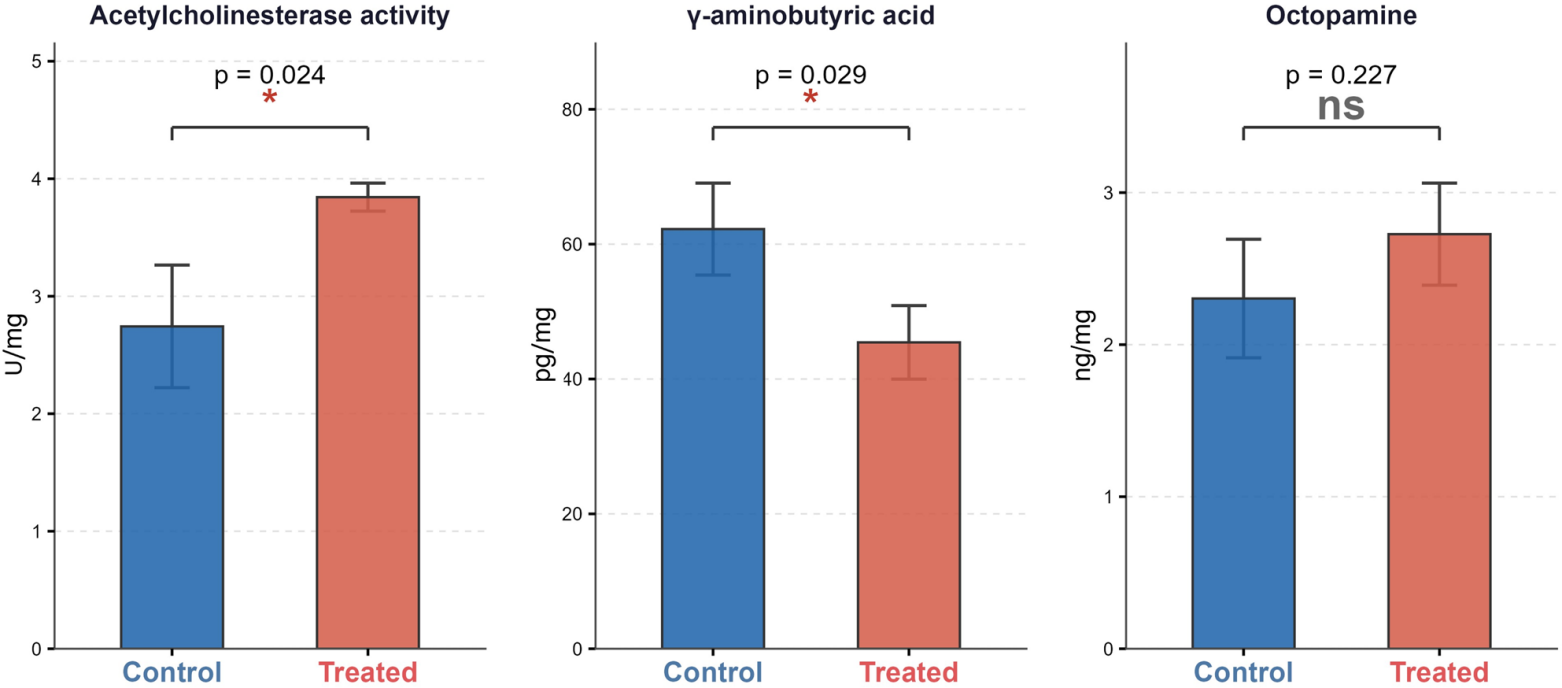
Quantitative levels of acetylcholinesterase activity (**A**), γ-aminobutyric acid (**B**), and octopamine (**C**) in *Culex pipiens* larvae following exposure to a sublethal concentration (LC_40_, 48 h) of Egyptian *Matricaria chamomilla* essential oil. Data are presented as mean ± standard deviation. * Indicates statistically significant difference at (*p* < 0.05), whereas the ns symbol refers to a non-significant difference

The results indicated that the Egyptian German chamomile oil had a marked effect on the neural responses of *Cx.pipiens* larvae (Fig. 14A–C). A significant increase in AChE activity was detected in treated larvae compared with the control group (t = − 3.56, *p* = 0.0236; Fig. 14A). In contrast, GABA levels were significantly reduced in treated larvae (t = 3.33, *p* = 0.0290; Fig. 14B). Regarding octopamine levels showed an increasing trend following treatment; however, the difference was not statistically significant relative to the control group (t = − 1.43, *p* = 0.227; Fig. 14C).

## Discussion

This study presents a novel integrative computational workflow for identifying neuroactive EOs with selective toxicity against *Cx. pipiens* larvae. By combining large-scale in silico screening with toxicity prediction, the proposed strategy enabled the rapid prioritization of EO candidates with potential mosquitocidal activity while minimizing predicted vertebrate toxicity. In contrast to conventional empirical screening approaches, which are often labor-intensive and time-consuming, the current workflow facilitated the systematic evaluation of 5,183 EOs, reducing the dataset to 1,345 oils with predicted low vertebrate toxicity. Further filtering based on predicted anti-mosquito activity identified 51 high-priority candidates for downstream investigation.

To evaluate the predictive reliability of toxicity-based prioritization, a toxicity index was developed using ProTox-derived endpoints to compare the identified compounds. The lowest predicted toxicity was observed for cedryl acetate, longiborneol acetate, 3-methylbutyl 4-methylpentanoate, and glyceryl monooleate, consistent with external safety evidence such as GRAS status, RIFM evaluations, and their established use in food, fragrance, and pharmaceutical products [35–38]. In contrast, highly ranked toxic compounds such as warfarin and calcitriol are well-known hazardous agents with narrow safety margins. Warfarin is a potent anticoagulant with low LD50 and high toxicity risk; Calcitriol causes hypercalcemia in rats [39, 40].

The index also enabled classification of EOs into low- and high-risk groups. *Mentha suaveolens*, *Citrus aurantium amara*, and *Mentha × villosa* were consistently identified as low-risk oils, in agreement with reported LD50 values (> 2000 mg/kg), GRAS status, and previous RIFM/IFRA safety evaluations [41–43]. Conversely, *Origanum saccatum*, *Prunus armeniaca*, *Prunus dulcis*, and *Paeonia emodi* were classified as high-risk materials due to known toxic constituents such as phenolics and cyanogenic glycosides [44–46]. The strong agreement between index predictions and established toxicological data supports the reliability of the framework for early-stage risk assessment of natural products.

The IMT index further identified several EOs as highly active against mosquito neural targets, with *Juniperus mexicana* ranking among the most potent candidates. This prediction agrees with previous reports showing strong larvicidal activity of *Juniperus sp* and *Cupressus funebris* oils, with LC50 values below 50 ppm [47, 48]. Sesquiterpenes associated with *Juniperus* oils, including cedrol, thujopsene, and cedrene derivatives, have been linked to mosquitocidal activity, with reported LC50 values ranging from 11 to 18 ppm for cedro [49]. Likewise, γ-eudesmol demonstrated complete larval mortality at approximately 250–300 ppm [50], supporting the role of oxygenated sesquiterpenes in toxicity. Other highly ranked oils, such as *Leptospermum scoparium* (LC50 ≈ 12–48 ppm) [51] and *Curcuma longa* (LC50 ≈ 17–29 ppm) [52], also correspond well with previously reported larvicidal activity. *Eugenia uniflora* showed strong activity (LC50 ≈ 35.9 ppm) [53], while *Salvia desoleana* [54], *Lippia carviodora* [55], and *Bixa orellana* [56] consistently fall within the 30–300 ppm activity range. The agreement between docking-based ranking and experimental LC50 data supports the predictive reliability of the proposed index for identifying mosquito-active neurotoxic EOs.

Crucially, the consistent emergence of German chamomile oils across multiple global chemotypes strongly validated the selection of the local Egyptian *M. chamomilla* EO evaluated in the present study. Previous studies have also reported the insecticidal activity of chamomile oils against different mosquito species [57, 58], further supporting the rationale for selecting the Egyptian German chamomile EO in the present study. GC–MS analysis confirmed that oxygenated sesquiterpenes, particularly bisabolol derivatives, were the predominant constituents of this oil. Several studies have also highlighted the potent insecticidal and neuroactive properties of these compounds, with β-farnesene, germacrene D, and α-bisabolol oxide A specifically implicated in the toxicity of *M. chamomilla* EO against malaria and Zika virus vectors [57, 59–61].

To explore the mechanistic basis of this activity, in silico molecular docking was performed against four key mosquito neural targets: AChE, which mediates cholinergic neurotransmission; the GABA_R_, responsible for extra-synaptic signaling; the VGSC, essential for action potential propagation; and the OAR, a critical regulator of behaviour, metabolism, and neuromuscular function. Docking analyses revealed that oxygenated sesquiterpenes, particularly bisabolol oxide A and bisabolone oxide A, exhibited strong binding affinities for AChE and GABA_R_, providing a theoretical basis for the predicted neurotoxic and larvicidal activity of chamomile EO.

The computational predictions were subsequently validated through experimental assays, which confirmed the pronounced insecticidal activity of the Egyptian German chamomile EO. Larvicidal bioassays demonstrated complete mortality (100%) at 400 ppm, a concentration lower than those reported in the previous studies [62], highlighting the high potency of this oil. Behavioural analyses revealed pronounced hyperactivity, repetitive body coiling, and self-biting of the anal papillae, which are characteristic indicators of neurotoxic excitation and neuromuscular disruption. Similar neurobehavioral disturbances have been observed in mosquito larvae exposed to fungal metabolites [63]. Pathological examination further revealed severe morphological deformities, including increased separation between the head and thorax, structural damage to the siphon, tracheal deformation, lysis of internal tissues, and partial separation of the head capsule. Comparable alterations have been reported in previous studies. For example, Larvae exposed to peppercorn extracts exhibited hyperactivity, convulsions, and paralysis, along with shrinkage and deformation of the anal papillae [64]. An increased separation between the head and thorax was also observed in *Anopheles darlingi* larvae following exposure to diflubenzuron [65]. More recently, a synergistic combination of geranial and trans-cinnamaldehyde caused swelling of the anal papillae and deformation of the siphon in Aedes aegypti larvae [66].

The present study showed that *Matricaria chamomilla* essential oil induced marked histological and immunohistochemical alterations in *Cx. pipiens* larvae. Control larvae exhibited normal tissue organization, whereas treated larvae showed severe tissue destruction and loss of structural integrity in both head capsule and internal organs. Immunohistochemically, a strong Caspase-3 expression was observed in treated larvae compared to controls, indicating activation of apoptosis as a key mechanism of tissue damage. These observations were consistent with the results of [30], who demonstrated that neem oil caused pronounced anatomical damage in *Aedes* larvae, including disruption of key tissue structures. Similarly [67], reported that exposure of mosquito larvae to *Carum copticum* extract induced significant histopathological alterations. The strong Caspase-3 immunoreactivity observed in the present study further supports the activation of apoptotic pathways, which aligns with previous reports of [68], who highlighted that Caspase-3 is a reliable biomarker for detecting apoptosis in *Aedes* mosquitoes, where its increased expression is strongly associated with programmed cell death and tissue degeneration following exposure to insecticidal agents.

These alterations may result from the interference of the chamomile oil components with cholinergic transmission, oxidative stress, or disruption of neural signaling integrity. This was true as the physiological assessments in the present study demonstrated a marked elevation in AChE activity and a substantial reduction in GABA levels. These observations align with previous reports on the effects of German chamomile oil and its constituents on cholinergic and GABAergic systems [69–71]. Although molecular docking predicted favorable interactions between chamomile-derived compounds and AChE, the observed increase in enzymatic activity suggests that these in silico interactions do not necessarily translate into functional enzyme inhibition under in vivo conditions. This apparent discrepancy may reflect the complexity of physiological responses in insect larvae, where neurophysiological compensation, stress-induced regulatory pathways, and oxidative stress can collectively modulate enzyme activity [72, 73]. Exposure to neuroactive phytochemicals may trigger adaptive upregulation of AChE as a protective response to maintain cholinergic homeostasis following transient or reversible disruption of neurotransmission. Similar compensatory enzymatic responses have been previously reported in insects exposed to botanical insecticides and neuroactive plant secondary metabolites [74]. Therefore, AChE activity modulation in the present study is likely multifactorial rather than solely determined by direct ligand–enzyme interactions predicted through molecular docking.

The strong concordance between in silico predictions and experimental findings provides compelling mechanistic evidence for the neuroactive and insecticidal properties of the Egyptian German chamomile EO. This study highlights the effectiveness of integrating molecular docking and chemoinformatic screening to guide the selection of bioactive phytochemicals for vector control. By combining these computational techniques, the selection process for promising botanical insecticides can be significantly accelerated while reducing time, resources, and experimental workload, as well as supporting more efficient and ethically responsible research practice. Nevertheless, certain limitations should be acknowledged. ProTox-II predictions provide relative toxicity estimates rather than absolute toxicity values and may show reduced accuracy when applied to chemically complex EOs. In addition, although the AlphaFold-generated protein structures demonstrated high confidence scores, they still require full experimental validation, particularly for neural targets.

## Conclusions

This study demonstrates that a tiered in silico screening framework is an effective and cost-efficient strategy for identifying promising plant-derived mosquito control agents and prioritizing candidates for experimental evaluation. Among the screened essential oils, German chamomile (*Matricaria chamomilla*) essential oil showed strong potential through both computational and experimental validation, exhibiting significant larvicidal activity and clear neurotoxic effects on *Culex pipiens* larvae. These findings highlight the value of integrating computational approaches with biological assays to accelerate the discovery of botanically derived alternatives to conventional insecticides and support the development of environmentally friendly vector control strategies. Further field-based validation and formulation studies are necessary to advance practical applications. Future studies should experimentally validate the most promising essential oils and their major constituents under laboratory and semi-field conditions. Additional molecular dynamics simulations and enzyme inhibition assays are recommended to provide deeper insight into their mechanisms of action. Furthermore, integrating computational screening with AI-based predictive models may accelerate the identification of effective and environmentally safe mosquito control agents.

## Supplementary Information

Below is the link to the electronic supplementary material.

Supplementary Material 1: Additional file 1: A detailed list of all essential oils, along with their corresponding compounds and relative abundance.

Supplementary Material 2: Additional file 2: Detailed profiles of the selected low-toxicity essential oils, including their constituent compounds and final toxicity scores

Supplementary Material 3: Additional file 3: Top 50 docked phytochemical compounds identified for each mosquito neural targets: acetylcholinesterase (AChE), GABA receptor (GABAR), octopamine receptor (OAR), and voltage-gated sodium channel (VGSC) based on binding affinity values (kcal•mol⁻¹).

Supplementary Material 4: Additional file 4: Complete ranking of Integrated Mosquito Target Index (IMT) for all assessed essential oils.

## Data Availability

The data used in this study are available from the corresponding author upon reasonable request.
